# Karyotype depends on sperm head morphology in some amniote groups

**DOI:** 10.3389/fgene.2024.1396530

**Published:** 2024-06-06

**Authors:** Eric M. Kramer, Joshua Enelamah, Hao Fang, P. A. Tayjasanant

**Affiliations:** ^1^ Department of Physics, Bard College at Simon’s Rock, Great Barrington, MA, United States; ^2^ Department of Biology, Bard College at Simon’s Rock, Great Barrington, MA, United States

**Keywords:** karyotype evolution, spermiogenesis, chromosome packaging, microchromosomes, amniotes, spermatozoa

## Abstract

The karyotype of an organism is the set of gross features that characterize the way the genome is packaged into separate chromosomes. It has been known for decades that different taxonomic groups often have distinct karyotypic features, but whether selective forces act to maintain these differences over evolutionary timescales is an open question. In this paper we analyze a database of karyotype features and sperm head morphology in 103 mammal species with spatulate sperm heads and 90 sauropsid species (birds and non-avian reptiles) with vermiform heads. We find that mammal species with a larger head area have more chromosomes, while sauropsid species with longer heads have a wider range of chromosome lengths. These results remain significant after controlling for genome size, so sperm head morphology is the relevant variable. This suggest that post-copulatory sexual selection, by acting on sperm head shape, can influence genome architecture.

## 1 Introduction

The karyotype of a species is the set of gross features of the chromosome complement, including their number and size, the location of centromeres, and the banding patterns visible after staining treatments (Matthey, 1949; Ohno, 1970; White, 1973). Karyotypic features are discernible with an optical microscope during mitosis or meiosis, although they can be readily evaluated using modern genomic methods as well. In the middle of the 20th century, as the number of published karyotypes rose into the hundreds, it became apparent that different taxonomic groups often show distinct karyotypic features. Perhaps the most familiar example of this is the fact that many groups have a diploid chromosome number (2n) that falls in a narrow range [see, e.g., (Oguiura et al., 2009; Degrandi et al., 2020)].

Another karyotypic feature that first attracted attention in the 20th century was the discovery of “microchromosomes”—chromosomes ≲1 μm in length at mitosis, small enough that their banding patterns and centromeres cannot be optically resolved (Srikulnath et al., 2021). Microchromosomes are autosomes, which means they occur as homologous pairs in somatic cells, segregate normally at meiosis, and are transmitted from parent to offspring via the germ cells. They are thus readily distinguished from other categories of small chromosomes: B-chromosomes, which never occur as homologous pairs (Vujosevic et al., 2018), and extrachromosomal bodies (ecDNA), which arise due to DNA damage in cancer cells and rarely enter the germ line (Verhaak et al., 2019). Among amniotes, microchromosomes are generally absent from mammalian karyotypes, while most species of birds and nonavian reptiles have multiple pairs, sometimes 10 or more (Tegelstrom et al., 1983; Oguiura et al., 2009; Degrandi et al., 2020). As early as 1975, this was recognized as a puzzle (Matthey, 1975; White, 1975). Since large-scale chromosome rearrangements, including fissions and fusions, are frequent over evolutionary time scales (Damas et al., 2018; Damas et al., 2022), the persistence of a whole category of small chromosomes in some lineages but not others would seem to require an explanation.

The modern era has seen progress on some aspects of this topic but not others. A recent comparison of genomes among the three amniote classes—Mammalia, Aves, and Reptilia—reveals that some microchromosomes in birds and non-avian reptiles have persisted as syntenic blocks throughout the evolution of those lineages (Waters et al., 2021; Damas et al., 2022). In mammals, which diverged from other amniotes ∼320 Mya, the ancestral microchromosomes have been lost via fusion with each other, or with larger macrochromosomes. Modern techniques have also shed light on the molecular aspects of microchromosomes. They are gene-rich, have a relatively high GC content, and are typically <30 Mb in length (Srikulnath et al., 2021). On the other hand, there is no unique genetic or biochemical marker for microchromosomes. They are simply small compared to those typically found in mammals.

While some molecular and evolutionary aspects of microchromosomes have been clarified, there is still no explanation for why they are common in Aves and Reptilia (collectively called sauropsids) but not in mammals. One plausible explanation is that they offer some adaptive advantage in sauropsids. This hypothesis has recently been tested by Mezzasalma et al. (Mezzasalma et al., 2023) in chameleons (family Chamaeleonidae). They examined 83 species with diploid chromosome numbers ranging from 2n = 20 to 62. The numerical differences were largely due to variation in the number of microchromosome pairs, and phylogenetic analysis suggested that chameleon species with fewer than 2n = 36 chromosomes had evolved independently several times, in each case due to microchromosome fusions. The authors then tested for a correlation between microchromosome loss in these chameleons and nine different ecological and life-history traits. No significant correlations were found, leading the authors to speculate that a tendency for microchromosome fusion may be “intrinsic” to the ancestral chameleon genome. Thus, the forces shaping observed karyotypic differences in amniotes remain poorly understood (Srikulnath et al., 2021).

**FIGURE 1:**
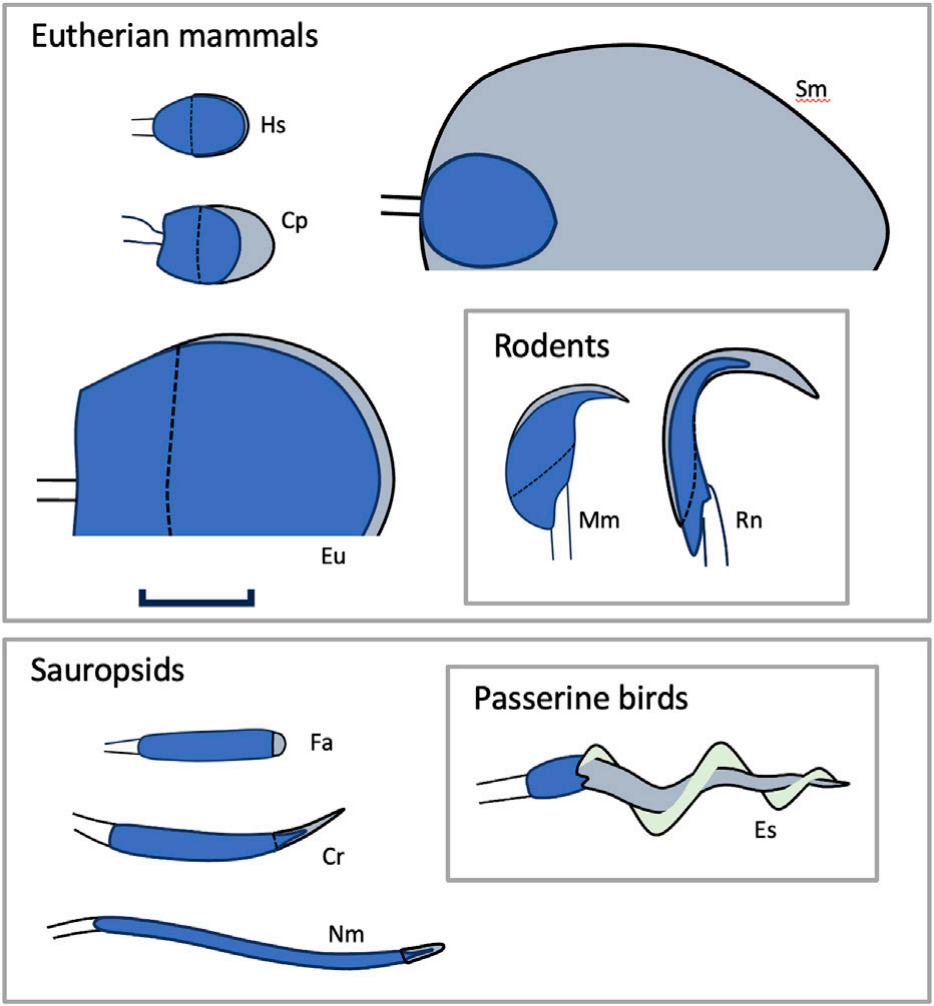
Sperm head morphology in amniotes. The figure illustrates some of the variation found in the morphology and size of sperm heads in amniotes. All figures to scale (scale bar 5 um). Blue: nucleus; gray: acrosomal complex; light green: the helical membrane that wraps the acrosomes of passerine birds. Dashed lines indicate the proximal extent of the acrosome. Solid lines distal to the heads indicate junction with the spermatozoon midpiece. Abbreviations: Cp = *Carollia perspicillata* (a phyllostomid bat), Cr = *Crotalus* sp. (rattlesnake), Es = *Emberiza schoeniclus* (reed bunting), Eu = *Euphractus sexcinctus* (an armadillo), Fa = Falco sp. (falcon), Hs = *Homo sapiens*, Mm = *Mus musculus* (mouse), Nm = *Numida meleagris* (a guineafowl), Rn = *Rattus norvegicus* (rat), and Sm = *Suncus murinus* (a shrew). Mammal nuclei are somewhat larger than sauropsid nuclei because they have larger genomes. The much larger size of the *E. Sexcinctus* nucleus does not indicate a correspondingly larger genome (it has 4.16 pg of DNA *versus*, for example, 3.5 pg in humans (Gregory, 2019)). Rather, the nucleus of Eu has a large area in projection because it is exceptionally thin, only ∼0.1 μm (Cetica et al., 1997). Sketches after references (Green and Dryden, 1976; Forman and Genoways, 1979; Cetica et al., 1997; Kohn et al., 1997; Breed, 2005; Jamieson, 2006; Jamieson, 2006; Medarde et al., 2013; Stostad et al., 2018; SDZWA, 2023) respectively.

In this paper we present evidence that selection does act on the karyotype in some amniote groups, indirectly, through its influence on sperm head morphology. The sperm head of amniotes lacks most of the familiar cytoplasmic compartments and is divided into two main parts: the acrosomal complex and the nucleus (Skinner and Johnson, 2017) (Figure 1). The acrosomal complex is a distally located, Golgi-derived structure that allows the sperm to fuse with the ovum. The nucleus has properties that are distinct from those at interphase, including a smaller volume and higher DNA density (Pogany et al., 1981; Perreault et al., 1988; Zakhidov et al., 2013). In spermatozoan nuclei, the majority of histones are replaced by smaller proteins called protamines that bind tightly to one another, turning the nucleus into an elastically rigid object. The mature sperm cell thus represents one of the few instances in the life of the organism where the nucleus makes a direct, physical contribution to the overall shape of the cell.

The morphology of sperm cells shows a remarkable variation between taxonomic groups (Figure 1)—perhaps more variation than any other cell type known (Roldan et al., 1992; Pitnick et al., 2009). Among mammal groups, sperm head morphology varies widely (Roldan et al., 1992). A flattened, oval shape is common in many mammalian orders, but it is not universal. In rodents, sperm heads may be hooked or adopt more elaborate shapes, and in monotremes the head is shaped like a helical thread 40–50 μm long (Carrick and Hughes, 1982). In sauropsids, sperm heads are generally narrow (diameter ∼1 μm) with a cylindrical, conical, or helical form (Jamieson, 1995b; Jamieson, 2006).

Although the evolution of such diverse sperm head shapes is still poorly understood, the consensus is that the various forms have been shaped in part by post-copulatory sexual selection (Snook, 2005; Pizzari and Parker, 2009; Fitzpatrick and Lupold, 2014). The function of the spermatozoon is to transport the haploid genetic complement of the male to the female. The sperm cell must successfully navigate the biochemical and physical environment of the female reproductive system, outcompete the sperm of rival males (in species where females mate with more than one male), remain viable during sperm storage within the female (common in sauropsids), and efficiently fuse with the ovum. Each of these tasks may impose distinct constraints on sperm morphology. The connection between sperm morphology and sexual selection is a topic of much current research, and there are many open questions. However, the selective advantage of some head shapes is well-established. For example, the hooked shape of rodent sperm allows them to form tangled aggregates that swim faster than singular sperm (Moore et al., 2002; Immler et al., 2007).

## 2 Methods

### 2.1 Data collection

We compiled a database of sperm head dimensions, genome size, chromosome number, and chromosome lengths at mitosis for amniote species with either spatulate or vermiform sperm heads. We began our data collection process with the available reviews of sperm morphology [e.g., (Roldan et al., 1992; Jamieson, 1995b; Jamieson, 2006)], and with the SpermTree database, v 01-21-22, available at spermtree.org (Fitzpatrick et al., 2022). For taxonomic groups of special interest not covered by the reviews, we conducted additional searches genus-by-genus using Google scholar (scholar.google.com) and Web of Science (Clarivate, London). Once we identified a species whose sperm morphology was available, we searched for a matching karyotype using the same resources. Genome DNA content, sometimes called the C-value, was mostly drawn from the database of Gregory (Gregory, 2019) and the NCBI portal (NCBI, 2023). Sperm morphology and karyotype data were tabulated for 193 species (see Supplementary Material). Genome size was available for 142 of the 193 species.

In many cases, sperm head parameters or chromosome lengths were available in the text of the paper. In the remaining cases these features were measured directly from figures. Images were imported into Fiji v 2.14.0 (Schindelin et al., 2012) and lengths were measured by hand using the segmented line tool.

### 2.2 Sperm head dimensions

Multiple authors report that electron microscopy measurements give sperm head dimensions that are smaller by ∼1 μm than those found using optical microscopy techniques (van der Horst et al., 1991; Stelzer et al., 2009; Villaverde-Morcillo et al., 2015; Kimsakulvech and Suttiyotin, 2020). To compensate for this effect, we add a correction of 1.0 μm to all head lengths, and to mammal widths, collected using scanning or transmission electron microscopy (SEM/TEM).

Sperm head length ℓ and width *w* are often tabulated in papers, but projected area is much less commonly found. For all mammal species, we estimate the projected sperm head area using the equation A = (π/4) ℓ*w*. This equation is exact for an ellipse, and it is also exact for an ellipse cut in half along its minor axis (giving a bullet shape). We also checked its predictions against published sperm head areas for human (Bellastella et al., 2010), bull (Beletti et al., 2005), and pig (Saravia et al., 2007) and found agreement within ∼10%.

### 2.3 Regressions

*Chromosome length*. To quantify the relationship between the shortest autosome length L_min_ and the dispersity of chromosome lengths K (defined K = L_max_/L_min_, discussed more fully in the Results section), the haploid chromosome number n, and the haploid genome size C, we performed a linear regression on the log-transformed data in Excel (Microsoft, v16.78). The regression equation

$$\log_{10}\left(L_{\min}\right)=\delta +\alpha \log_{10}\left(C\right)+\beta \log_{10}\left(K\right)+\gamma \log_{10}\left(n\right)$$ (1)

can then be re-written as a power-law relationship, L_min_ = D(C)^α^(K)^β^(n)*^γ^*, where the constant D = 10^δ^ and the other parameters appear as exponents. We used ordinary least squares for L_min_, rather than the phylogenetic approach used for the rest of our analyses (see below) because we are interested in the shape of the “niche space” as described by Price (Price, 1997).

*Karyotype features*. Because the evolutionary relationship between species can lead to correlations, especially among recently diverged pairs of species, treating the traits of each species in the database as statistically independent can lead to false positive significance tests in ordinary least squares (Felsenstein, 1985). Phylogenetic generalized least squares (PGLS) is a family of methods designed to account for the effects of species relatedness in regression models. Specifically, we used the PGLS technique of Pagel’s λ (Pagel, 1999; Revell and Harmon, 2022), which estimates the importance of “phylogenetic inertia” to any observed trend and provides improved estimates for the fit parameters and their standard errors. The importance of phylogeny is characterized by a phenomenological parameter λ, which falls between λ = 0 (no phylogenetic effect) and λ = 1 (substantial phylogenetic effect). Software packages that calculate Pagel’s λ estimate it using a maximum likelihood technique. Following the treatment in (Revell and Harmon, 2022), we perform our analysis in R v 4.3.2 (R Core Team, 2018) using the packages ape v 5.7 (Paradis and Schliep, 2019), geiger v 2.0.11 (Pennell et al., 2014), nlme v 3.1 (Pinheiro et al., 2023), and phytools 2.0-3 (Revell, 2024).

Our use of PGLS techniques required phylogenetic trees for the Mammalia and Sauropsida species in our database. These were generated using TimeTree v 5 (Kumar et al., 2022). Additional details, and plots of the phylogenies, may be found in the Supplementary Material.

To evaluate the dependence of karyotype on gross sperm head morphology, we introduced a parameter s, which equals 1 for vermiform sauropsid sperm heads and 0 for spatulate mammalian sperm heads. Then, we fit the equations log_10_(n) = α + β s and log_10_(K) = α + β s. A value of β significantly different from 0 would correspond to a difference between sauropsid and mammal karyotypes that cannot be explained by random drift over evolutionary timescales.

To quantify the dependence of karyotype parameters n and K on sperm dimensions and genome size, we performed a regression on the log-log transformed data. The model dependence of karyotype feature y on independent variable x is then log_10_(y) = α + β log_10_(x). This can be re-written as a power-law relationship y = A(x)^β^, where the constant A = 10^α^ and β appears as an exponent.

## 3 Results

### 3.1 Survey of amniotes

In this paper we limit consideration to amniote species. Sperm morphology and morphogenesis has been thoroughly studied in amniotes, and they share many features not found in anamniotes (Yoshida, 2016). In addition, the karyotypes of amniotes have been studied for more than a century, and thousands of species have been characterized [e.g., (Becak et al., 1971; O’Brien et al., 2020)]. Thus, amniotes are well-suited to an examination of the relationship between karyotype and sperm morphology.

**FIGURE 2:**
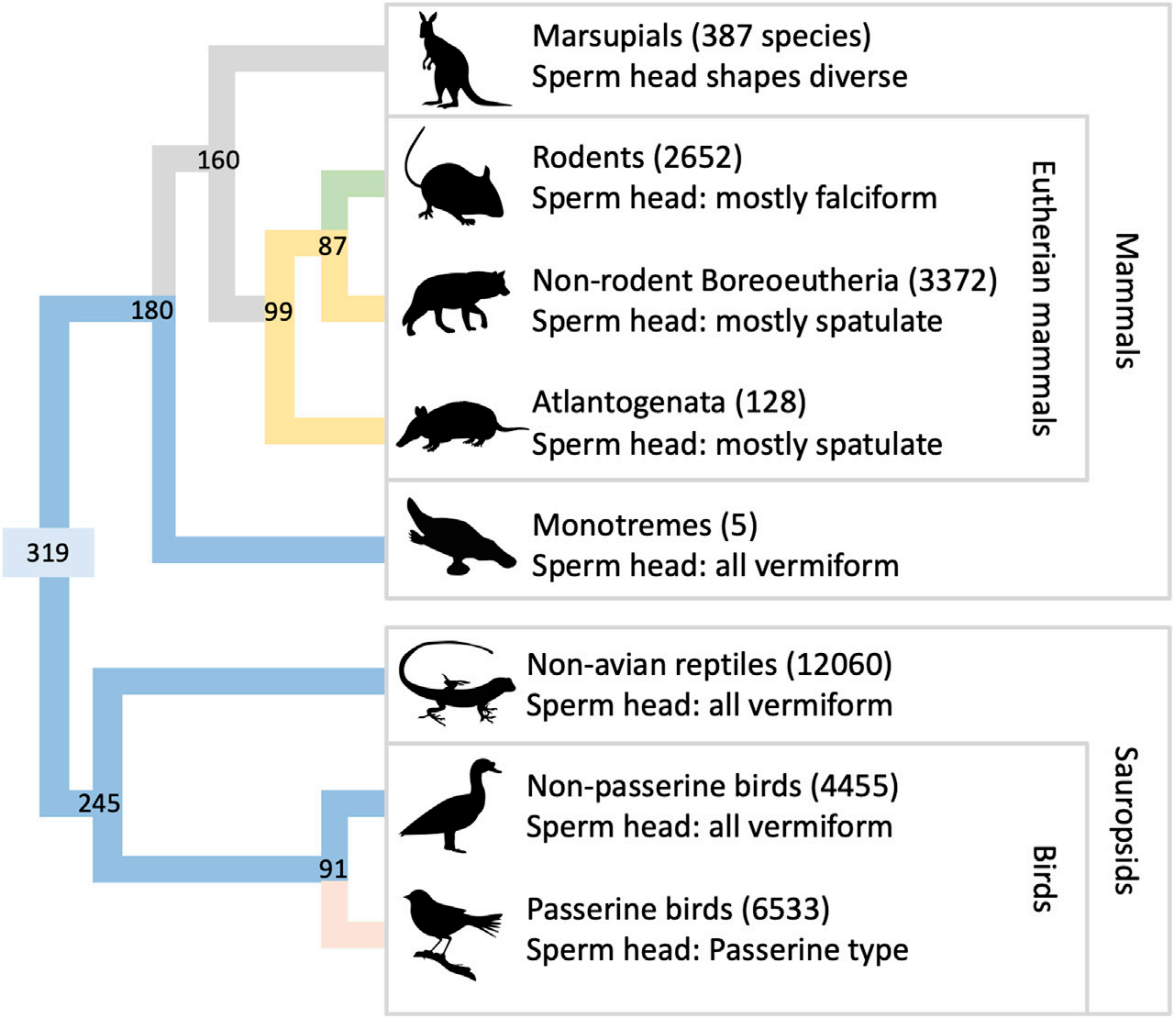
Phylogeny of amniote species. The figure shows the relationships among extant taxonomic groups of amniotes, arranged to clarify evolutionary aspects of sperm head morphology. Numbers next to group names are species totals (Gill et al., 2023; MDD, 2023; Uetz et al., 2023). The phylogenetic tree at left shows divergence dates at each node (units Mya; branch lengths not to scale), generated using TimeTree v 5.0 (Kumar et al., 2022). The color assigned to terminal branches indicates the most common sperm morphology in each group (Roldan et al., 1992; Jamieson, 1995b; Jamieson, 2006; Friesen et al., 2020): Green is majority falciform, yellow is spatulate, blue is vermiform, pink is passerine-type, and gray indicates branches with a diversity of forms and/or unresolved history. Color is assigned to older branches on the hypothesis that the spatulate head shape and the vermiform head shape each had a single, ancestral origin event.

A compressed phylogeny of amniotes is shown in Figure 2, illustrating the relationship among the groups discussed in this paper (Kumar et al., 2022). Modern amniotes are divided into Mammalia and Sauropsida, which diverged ∼319 Mya. Mammals are further divided into monotremes, marsupials, and eutherian mammals. Eutherian mammals split into Atlantogenata and Boreoeutheria ∼99 Mya. Sauropsids are divided into the taxonomic groups Aves and Reptilia, which diverged ∼245 Mya. The sperm heads of most sauropsids (excepting the speciose order of passerine birds) share three traits in common: an elongated nucleus, approximately circular in cross-section, and a relatively small acrosome. For brevity we will describe heads with this set of traits as “vermiform”. Vermiform heads are also found in monotreme species, which suggests that this morphology may have characterized the earliest mammals (Jamieson, 1995a). The sperm heads of many eutherian mammals also have three traits in common: in projection, the nucleus is rounded, especially along its leading edge; in transverse section the nucleus appears flattened; and the acrosome matches these features in that it is also flattened and has a rounded leading edge. We will refer to heads with this set of traits as “spatulate”. Spatulate heads are not universal among eutherian mammals, and exceptions may be found in many orders (see the Supplementary Material for more discussion). Most notable among the exceptions is order Rodentia, whose species generally have a hooked sperm head shape (Figure 1). Since Boreoeutheria and Atlantogenata both have many species with spatulate sperm heads, it is likely that this set of traits also characterized their common ancestor (99 Mya). Marsupials, on the other hand, do not share any of these traits (Temple-Smith, 1994). Thus, it is likely that spatulate heads evolved sometime after eutherian mammals diverged from marsupials, 160 Mya.

The fact that most sauropsids have an elongated sperm head, while this feature is absent from eutherian mammals, combined with the already-noted puzzle about microchromosome abundance, was the original inspiration for this research. The question was whether this anecdotal observation hinted at an underlying quantitative relationship. To make progress, we decided to restrict consideration to spatulate and vermiform sperm heads, as these two morphologies are simple enough to quantify with just one or two clearly defined measurements (head length and width). Although thickness data would have permitted us to make direct estimates of sperm head volume, the smallest dimension of spatulate and vermiform heads are rarely known with sufficient accuracy to be useful (van Duijn, 1971; Stelzer et al., 2009; Villaverde-Morcillo et al., 2015).

Most spatulate and vermiform heads have relatively small acrosomes, in which case head dimensions are a reasonable proxy for nuclear dimensions. To ensure this correspondence between head and nuclear morphology, we excluded species where the nucleus did not occupy at least 50% of the total head length (see the Supplementary Material for more discussion of excluded groups).

### 3.2 Database

We collected data on karyotype and sperm head dimensions in 193 amniote species. Some summary statistics describing the database are presented in Supplementary Table S1. The number of species in our database is only 1%-2% of the amniote total (Gill et al., 2023; MDD, 2023; Uetz et al., 2023)—but we made every effort to cover the full range of diversity in karyotype and head size. For example, the database includes *Muntiacus muntjak* (Indian muntjac), which has the lowest known chromosome number of any mammal (2n = 6), and *Tympanoctomys barrerae* (red viscacha rat), which has the highest (2n = 102).

### 3.3 Karyotype parameters

**FIGURE 3:**
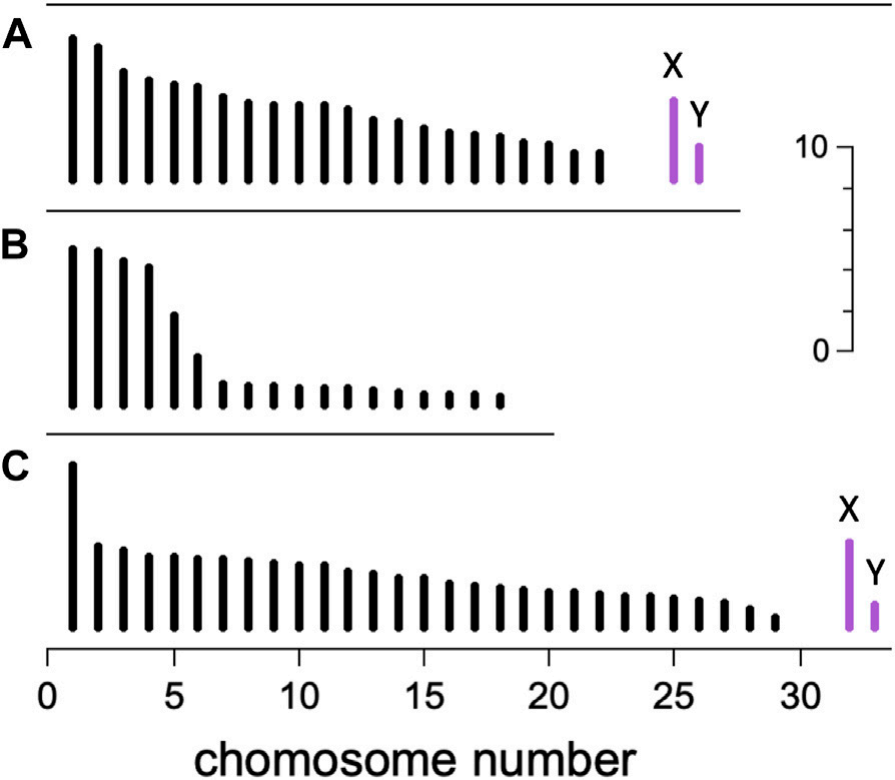
Karyotypes. Schematic illustration showing the length of mitotic chromosomes in three amniote species. Autosomes (black) are numbered from longest to shortest, and the heteromorphic XY pair of sex chromosomes (violet, not present in **(B)**) appear at right. Scale bar is 10 μm. **(A)** *Homo sapiens* (2n = 46) (Harnden and Klinger, 1985; Kramer et al., 2021) shows a typical eutherian mammal karyotype, with gradually decreasing chromosome lengths. **(B)** *Tropidurus torquatus* (an iguanid lizard, 2n = 36) (Becak et al., 1972) shows an abrupt transition between macro- and microchromosomes typical of sauropsids. **(C)** *Eulemur fulvus* (brown lemur, 2n = 60) (Hamilton and Buettner-Janusch, 1977) has a karyotype with two unusual features for a mammal: a large length transition between chromosomes 1 and 2, and a small chromosome 29, only ∼0.5 μm long, that qualifies as a microchromosome. The chromosome dispersity K is defined to be the length ratio of longest to shortest autosomes. Top to bottom, K = 5.4, 18.5, and 14.6.

Figure 3 illustrates some features of amniote karyotypes. The karyotype of *Homo sapiens* is typical of eutherian mammals, in that the chromosomes show an approximately linear decrease in size from longest to shortest. The karyotype of the lizard *Tropidurus torquatus* is typical of sauropsids, in that there appear to be two cohorts of chromosomes—a long and a short set—separated by a length “gap”. It was observations of karyotypes like this that initially suggested chromosomes might be divided into macro- and microchromosome categories. It should be noted that there are numerous exceptions to these general trends among both mammals and sauropsids (Matthey, 1975).

For our analysis, we needed to quantify the karyotype with one or more parameters related to the presence of microchromosomes. One obvious choice was the haploid chromosome number n. In karyotypes without a notable length gap we expected larger n to correspond with smaller chromosomes (Kramer et al., 2021). However, karyotypes with a length gap (e.g., Figures 3B, C) might confound this expectation. Since metaphase chromosomes contract throughout mitosis (Yunis et al., 1978; Van Dyke et al., 1986), the use of an absolute length cutoff to delineate microchromosomes would yield counts and sizes that depend on the degree of contraction. On the other hand, the relative lengths of chromosomes are known to remain approximately constant during contraction. To take advantage of this constancy, we chose to introduce a new quantity called the chromosome *dispersity*, defined K = L_max_/L_min_, where L_max_ and L_min_ are respectively the longest and shortest autosome lengths, measured during mitosis. We focused on autosomes to avoid complications from both heteromorphic sex chromosomes and supernumerary “B” chromosomes, which are often too small to measure precisely. Since the length of mitotic chromosomes is approximately proportional to their DNA content (Mayall et al., 1984; Praca-Fontes et al., 2014), the dispersity is a feature of the genome itself, not just a feature of cells in mitosis. The principal disadvantage of K is the reliance on L_min_, a value that will be imprecisely known in cases where the smallest chromosomes are less than about 0.5 μm.

To test the relative importance of chromosome number n, dispersity K, and overall genome size C for the occurrence of microchromosomes, we performed a simultaneous regression of L_min_ on all three quantities, L_min_ = D(C)^α^(K)^β^(n)*^γ^*. This revealed that L_min_ depends significantly on both K and n, with exponents β = −0.63 ± 0.10 and *γ* = −0.57 ± 0.11 respectively (N = 65, *R* ^2^ = 0.72, significance by 2-sided *t*-test, *p* < .05; remaining fit parameters may be found in the Supplementary Material). As expected, high chromosome numbers and high dispersity both correspond with smaller L_min_ values. The high value of *R* ^2^ shows that n and K together are good predictors of small chromosome size.

### 3.4 Karyotype and sperm morphology

We next sought to examine whether either karyotype parameter—haploid number n or dispersity K—depended significantly on a range of possible factors. Any such regression must control for the evolutionary history of species, since closely related species will be expected to have similar karyotypes, genome sizes, etc (Felsenstein, 1985). As detailed in the Methods section, the class of techniques used to control for relatedness are called phylogenetic generalized least squares (PGLS). In addition to lists of values for the characters being compared, PGLS techniques require as input a phylogenetic tree for all species in the analysis. Throughout our investigation we used the PGLS technique of Pagel’s λ (Pagel, 1999; Revell and Harmon, 2022), and phylogenetic trees generated by TimeTree v 5 (Kumar et al., 2022). Although our analysis was limited to just two paraphyletic groups of amniotes, Pagel’s λ analysis is robust against the presence of gaps in phylogenies (Molina-Venegas and Rodríguez, 2017).

We began by using Pagel’s λ to test for a dependence of karyotype (n or K) on genome size using the 142 species in the database for which a genome size was available. This analysis found no significant trends (regression parameters in Supplementary Material). We next examined whether karyotype (n or K) differed significantly between the two head shapes (Methods and Supplementary Material—see discussion of the parameter s, *N* = 193 species). This also found a negative result.

**FIGURE 4:**
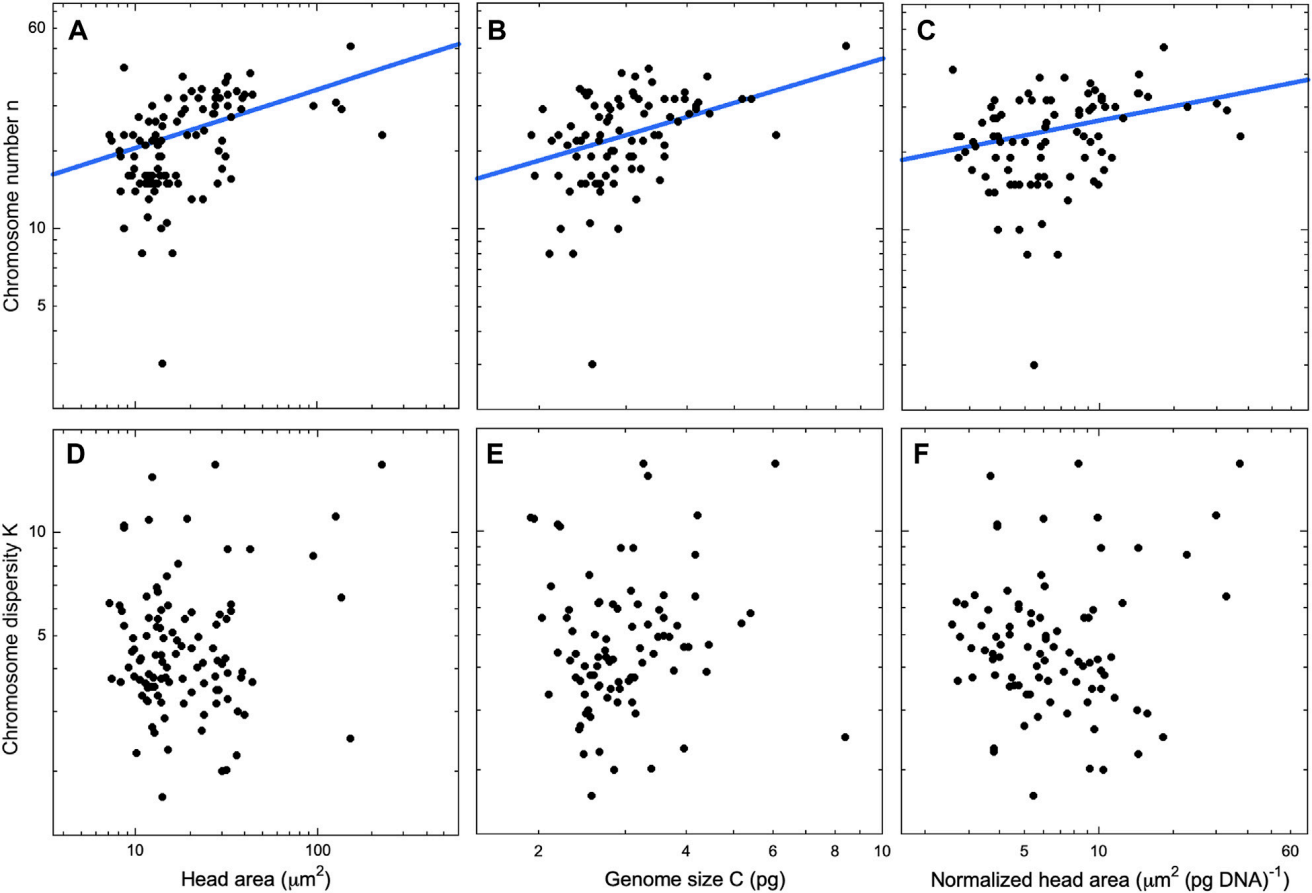
Mammals with spatulate sperm heads. Chromosome number (top row) and chromosome dispersity (bottom) row as a function of sperm head area **(A,D)**, genome size **(B,E)**, and head area per pg of DNA **(C,F)**. Only regression lines with exponents significantly different from 0 are shown [2-sided *t*-test, *p* < .05, fits using the PGLS technique Pagel’s λ (Pagel, 1999)]. The exponents are **(A)** β = 0.23 ± 0.07 (λ = 0.33, *R* ^2^ = 0.122) **(B)** β = 0.56 ± 0.21 (λ = 0.21, *R* ^2^ = 0.170), and **(C)** β = 0.19 ± 0.08 (λ = 0.30, *R* ^2^ = 0.026). Our database includes head area data for N = 103 species but genome size for only N = 87 species. Thus, the regressions in **(A)** and **(D)** include more data points than those in the other panels. Deletion of *Muntiacus muntjak* (Indian muntjac, 2n = 6) as a possible outlier, does not change the significance of the regressions. Additional data for all regressions may be found in the Supplementary Material.

**FIGURE 5:**
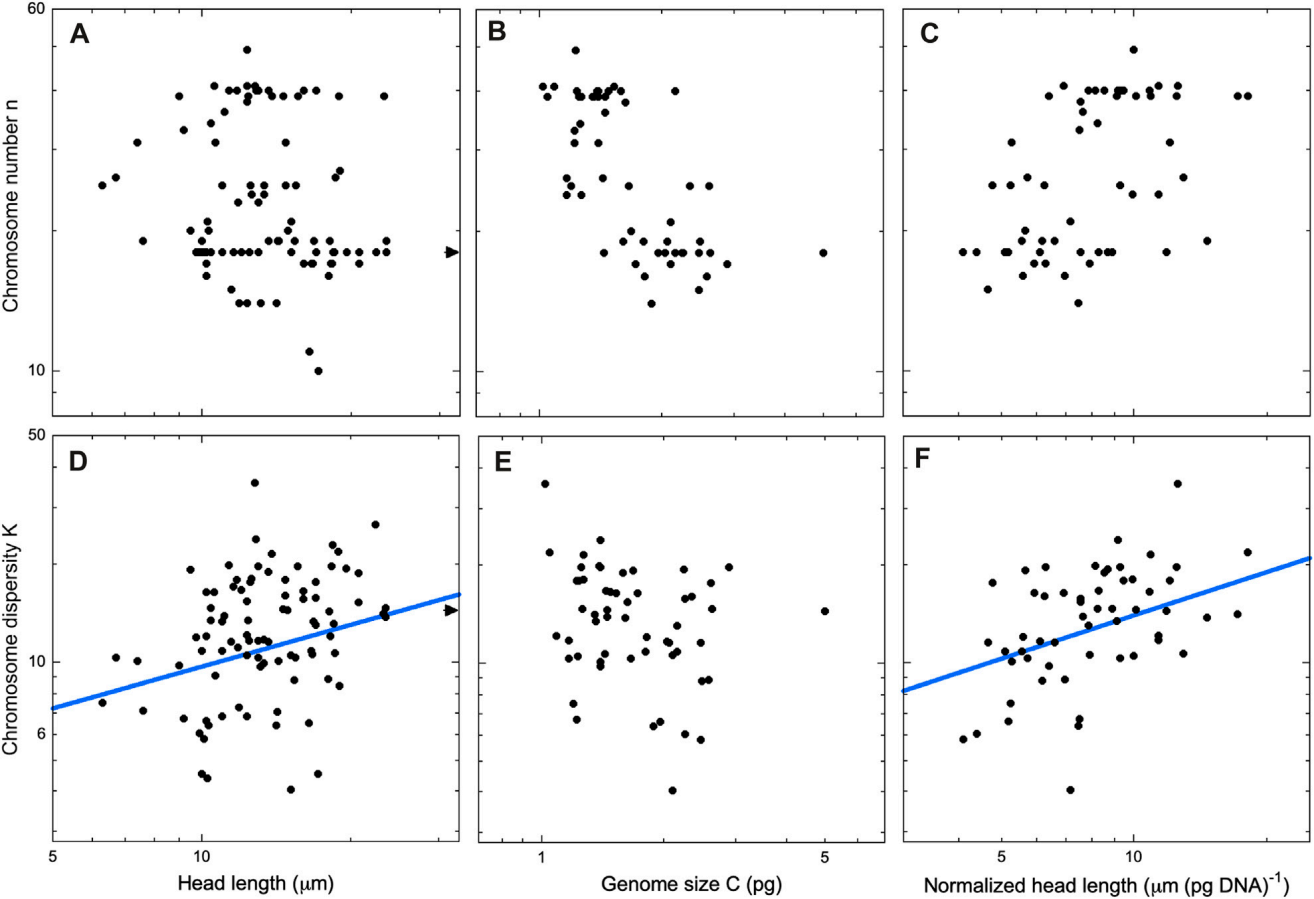
Sauropsids with vermiform sperm heads. Chromosome number (top row) and chromosome dispersity (bottom) row as a function of sperm head length **(A,D)**, genome size **(B,E)**, and head length per pg of DNA **(C,F)**. Only regression lines with exponents significantly different from 0 are shown [2-sided *t*-test, *p* < .05, fits using the PGLS technique Pagel’s λ (Pagel, 1999)]. The exponents are **(D)** β = 0.43 ± 0.17 (λ = 0.71, *R* ^2^ = 0.049) and **(F)** β = 0.45 ± 0.17 (λ = 0.30, R2 = 0.205). Our database includes head length data for N = 90 sauropsid species but genome size for only N = 55 species. Thus, the regressions in **(A)** and **(D)** include more data points than those in the other panels. Arrows in panels **(A)** and **(D)** indicate *Sphenodon punctatus* (tuatara, K = 14.4, ℓ = 59 μm), outside the borders of the figure. Deletion of *Sphenodon punctatus* as a possible outlier does not change the significance of the regressions. Additional data for all regressions may be found in the Supplementary Material.

Following these preliminary analyses, we decided to analyze spatulate and vermiform sperm heads as two separate cohorts. If n and K have distinct trends for the two head geometries, then a combined analysis might obscure the dependency. Indeed, we found such a disparity (Figures 4, 5, left panels). In spatulate mammals the chromosome number, but not the dispersity, increases significantly with head area. In vermiform sauropsid heads, the dispersity but not the number increases with head length (The reader may wonder whether chromosome number also tracks spatulate head length. We confirmed that the use of head length in place of area in mammals does not change the significance of the regressions. Details in Supplementary Material).

Anticipating that sperm head area or length might be serving as a proxy for genome size, we next did a regression of karyotype parameters against genome size (Figures 4, 5, center panels). In mammals n did indeed show a significant dependence on genome size. In sauropsids, by contrast, no trend was apparent. Although the dispersity K did not depend on C in sauropsids, it should be noted that the lack of genome data for 39% (35/90) of sauropsid species in the database would make any trend harder to detect.

For both spatulate and vermiform heads, we sought to control for the effects of genome size by introducing a “normalized” head dimension. That is, we divided head area or length by the genome size. After this normalization, both above-noted trends in n and K remained significant, and the values of the exponents remained positive (Figures 4, 5, right panels).

## 4 Discussion

We began our analysis with a regression of chromosome number on genome size for amniote species in the database (142/193 species have genome data available). This found no significant dependence. This result is especially notable since partial or whole genome duplications would tend to increase both variables simultaneously, so we might have expected a positive and significant dependence. The simplest explanation for the lack of a relationship appears to be that chromosome gains and losses are occurring at similar rates, sufficient to moderate any overall increase (Damas et al., 2018; Waters et al., 2021; Damas et al., 2022).

We then turned to sperm head morphology. Somewhat surprisingly, we found that the difference in gross morphology between spatulate and vermiform heads did not offer a significant explanation for the difference between mammal and sauropsid karyotypes. Thus, we decided to examine sauropsid and vermiform heads as two distinct groups, and to look for trends within each cohort separately. This approach was successful. On this topic, we can note the similar negative result of Gage (Gage, 1998). As part of a larger analysis of sperm dimensions in mammals, he found no relationship between chromosome number and sperm head length. Although it is unclear exactly which species were used in his analysis, his paper includes data for all major orders of mammals. Thus, it seems likely that his negative result is due, at least in part, to the use of a dataset that includes species with a wide diversity of head morphologies.

The main result of our paper is that chromosome number and dispersity show a significant dependence on head morphology, even after we control for genome size. In mammals with spatulate heads, chromosome number depends on projected head area. In sauropsids with vermiform heads, chromosome dispersity increases with head length. It is notable that the two head morphologies show contrasting behaviors. Since both n and K are significant predictors of microchromosomes in a karyotype, this suggests that small chromosomes might be generated via distinct processes in mammals and sauropsids.

To understand the possible mechanisms that might relate head morphology to karyotype, it will be helpful to first review spermiogenesis. Spermiogenesis is the late stage of spermatogenesis, where dramatic cell elongation takes place and the sperm cell takes on its mature form (Gribbins, 2011; Aire, 2014; de Boer et al., 2014). Spermiogenesis begins with a spermatid at the “round” stage—a haploid germ cell with an approximately isodiametric shape. As the spermatid elongates, it sheds cytoplasm, nuclear membrane, and nucleoplasm, and the nuclear volume decreases (Paci et al., 2018). The familiar histones of somatic cells are displaced from the DNA at this stage, to be replaced by a class of proteins called protamines that facilitate further nuclear compaction. This phase of spermiogenesis coincides with a relatively high frequency of DNA double-stranded breaks (DSB’s) (Leduc et al., 2008). Most DNA damage is repaired in the mature spermatozoa, but there is no post-meiotic checkpoint to ensure genome integrity. In humans, ∼10% of mature sperm retain damaged DNA (Irvine et al., 2000).

It is also during spermiogenesis that the spermatid nucleus transitions from a spheroidal shape to the flattened or elongated shape commonly found in the mature spermatozoon. Axes destined to become the thickness of a spatulate head, or the diameter of a vermiform head, experience very large strains, generally contracting by an order of magnitude. In humans, for example, the round spermatid nucleus is ∼10 μm in diameter (Paci et al., 2018), while the mature sperm head is less than ∼2 μm in thickness (van Duijn, 1960). We hypothesize that the degree of contraction at this stage contributes to the frequency and location of DSB’s, and in so doing influences the evolution of the karyotype.

Indeed, the normalized area introduced in the previous section offers a way to quantify the degree of contraction experienced by a spatulate nucleus during spermiogenesis. Assuming the density of sperm chromatin is similar across mammal species, then the mass of DNA per unit area is proportional to the mean nuclear thickness. A larger normalized area thus corresponds to a thinner head. A parallel argument can be made for the normalized length of vermiform heads.

While most DNA damage in spermatozoa simply leads to infertility, there is some evidence that it can contribute to changes in the karyotype of a lineage over evolutionary timescales. (Alvarez-Gonzalez et al., 2022) reconstructed the genomes of seven ancestral rodent species and identified evolutionary breakpoint regions (EBR’s) - genomic sites of ancestral chromosome breaks and rearrangements. The authors then compared these EBR’s to the locations of DSB’s most frequently observed in post-meiotic mouse spermatids. They found that EBRs tend to co-localize with a subset of DSB hotspots, and concluded that DNA damage in the male germ line provides the raw material for “evolutionary genome reshuffling”.

As for why mammals and sauropsid karyotypes might show the distinct trends reported here, we have no detailed hypothesis, but two observations may be relevant. First and most obvious is the gross difference in nuclear morphology—spatulate vs. vermiform. Second, we can point to studies of chromosome localization in the mature sperm head. In the sperm heads of eutherian mammals, chromosomes occupy globular or rod-shaped territories similar in size to mitotic chromosomes (Zalenskaya and Zalensky, 2004; Acloque et al., 2013; Chagin et al., 2018). In the sperm heads of sauropsids, by contrast, chromosomes can occupy threadlike territories that are an order of magnitude longer than their mitotic length (Solovei et al., 1998; Tsend-Ayush et al., 2009). In other words, the strains experienced by chromosomes during sauropsid spermiogenesis may be larger than in mammals because of group-specific differences in chromosome packaging. It seems likely that further insights will require a more detailed understanding of the coupling between chromosome breakage, sperm head morphogenesis, and the activity of DNA repair mechanisms.

## 5 Conclusion

Our results show that changes in amniote karyotypes over evolutionary timescales are at least partly constrained by sperm head morphology, but the underlying mechanisms remain unclear. The dynamics of chromatin during spermiogenesis is a topic of much ongoing research [e.g., (Ioannou et al., 2017; Alvarez-Gonzalez et al., 2022)], and we are optimistic that this work will help to clarify the functional connections. In addition, searches for similar correlations in groups other than amniotes, and over evolutionary history, may find additional, instructive examples.

These questions may also be clarified by a more detailed examination of the divergence of mammals and sauropsids, and the evolution of modern mammalian features. Genomic and phylogenetic analysis suggests that early mammals (∼180 Mya) were similar to modern sauropsids in terms of both karyotype and sperm head morphology (Jamieson, 1995a; Damas et al., 2022). However, by the time that eutherian mammals began their radiation into the lineages of Boreoeutheria and Atlantogenata, ∼100 Mya, spatulate head shapes and karyotypes lacking microchromosomes had both evolved. The degree to which changes in karyotype were synchronous with changes in head shape is a topic for future study. However, the fact that the loss of microchromosomes coincides at least approximately with the loss of the vermiform sperm head shape is consistent with our hypothesis.

Our results suggest a possible connection between two mechanisms of evolution previously regarded as independent: post-copulatory sexual selection and chromosomal evolution. Post-copulatory sexual selection encompasses a wide range of selection pressures on sperm morphology and physiology due to events that occur within the female reproductive tract (Snook, 2005; Pizzari and Parker, 2009; Fitzpatrick and Lupold, 2014). The most thoroughly studied example of this is sperm competition, but cryptic female choice can occur through a variety of proposed mechanisms. Chromosomal evolution is the idea that species divergence can be mediated in part by changes in karyotype, perhaps via changes in gene expression and a depression of hybrid fitness (Rieseberg, 2001). Both mechanisms of evolution are a focus of much ongoing research, and our study raises the possibility that they should not be considered independently.

## Data Availability

The data sets used in this study are openly available at the OSF data repository, under the title “Karyotype and Sperm Morphology”, https://osf.io/j45xs/.
