# Erratum: Karyotype depends on sperm head morphology in some amniote groups

**DOI:** 10.3389/fgene.2024.1462463

**Published:** 2024-07-19

**Authors:** Frontiers Production Office

**Affiliations:** Frontiers Media SA, Lausanne, Switzerland

**Keywords:** karyotype evolution, spermiogenesis, chromosome packaging, microchromosomes, amniotes, spermatozoa

Due to a production error, in subsection “**3.4 Karyotype and sperm morphology**,” paragraph 2, the following sentence was included incorrectly: “We next exaof seven ancestral rodent speciesmined whether karyotype (n or K) differed significantly between the two head shapes (Methods and Supplementary Material—see discussion of the parameter s, N genomes of seven ancestral rodent species and identified = 193 species).”

The correct sentence should read as:

“We next examined whether karyotype (n or K) differed significantly between the two head shapes (Methods and Supplementary Material—see discussion of the parameter s, N = 193 species).”

The publisher apologizes for this mistake.

The original version of this article has been updated.

