# Supplementary material for: Karyotype depends on sperm head morphology in some amniote groups
Source: Front Genet. 2024 Jun 6;15:1396530. doi: 10.3389/fgene.2024.1396530 (PMC11186999; doi:10.3389/fgene.2024.1396530)
Supplement: Supplementary file 1 [file DataSheet2.PDF]

Database References

Alföldi, J., Di Palma, F., Grabherr, M., Williams, C., Kong, L., Mauceli, E., et al. (2011). The genome of the green anole lizard and a comparative analysis with birds and mammals. *Nature* 477, 587–591. doi: 10.1038/nature10390

Altmanová, M., Rovatsos, M., Kratochvíl, L., and Johnson Pokorná, M. (2016). Minute Y chromosomes and karyotype evolution in Madagascan iguanas (Squamata: Iguania: Opluridae). *Biol. J. Linn. Soc.* 118, 618–633. doi: 10.1111/bij.12751

Andersen, K. (1973). Morphological and Ultrastructural Studies of Moose Spermatozoa. *Acta Vet Scand* 14, 81–91.

Anderson, M. J., Nyholt, J., and Dixson A.F. (2005). Sperm competition and the evolution of sperm midpiece volume in mammals. *Zool. Soc. Lond.* 267, 135–142. doi: 10.1017/S0952836905007284

Andraszek, K., Banaszewska, D., Szeleszczuk, O., Niedbala, P., and Kuchta-Gladysz, M. (2020). Comparison of the structure of chinchilla sperm isolated from semen and from the tail of the epididymis. *Reprod. Domest. Anim.* 55, 515–522. doi: 10.1111/rda.13646

Arnason, U., Benirschke, K., Mead, J. G., and Nichols, W. W. (1977). Banded karyotypes of three whales: Mesoplodon europaeus, M. carlhubbsi and Balaenoptera acutorostrata. *Hereditas* 87, 189–200. doi: 10.1111/j.1601-5223.1978.tb01262.x

Arnason, U., Sandholt, B., and Lutley, R. (1980). Banding studies on six killer whales: An account of C-band polymorphism and G-band patterns. *Cytogenet. Genome Res.* 28, 71–78.

Arslan, A., and Zima, J. (2014). Karyotypes of the mammals of Turkey and neighbouring regions: a review. *Folia Zool.* 63, 1–62. doi: 10.25225/fozo.v63.i1.a1.2014

Asa, C., Phillips, D. M., and Stover, J. (1986). Ultrastructure of Spermatozoa of the Crested Tinamou. *J. Ultrastruct. Mol. Struct. Res.* 94, 170–175. doi: 10.1016/0889-1605(86)90063-7

Aslam, H., Schneiders, A., Perett, M., Weinbauer, G. F., and Hodges, J. K. (2002). Quantitative assessment of testicular germ cell production and kinematic and morphometric parameters of ejaculated spermatozoa in the grey mouse lemur, Microcebus murinus. *Soc. Reprod. Fertil.* 123, 323–332. doi: 10.1530/rep.0.1230323

August, K., Davison, M., and Bortoluzzi, C. (2022). The genome sequence of the northern goshawk, Accipiter gentilis (Linnaeus, 1758). *Wellcome Open Res.* 7, 122. doi: 10.12688/wellcomeopenres.17821.1

Aula, P., and Kääriäinen, L. (1964). The Karyotype of the Elk (alces Alces). *Hereditas* 51, 274–278. doi: 10.1111/j.1601-5223.1964.tb01935.x

Baccetti, B., Burrini, A. G., and Falchetti, E. (1991). Spermatozoa and relationships in Palaeognath birds. *Biol Cell* 71, 209–216. doi: 10.1016/0248-4900(91)90067-w

Badenhorst, D., Hillier, L. W., Literman, R., Montiel, E. E., Radhakrishnan, S., Shen, Y., et al. (2015). Physical Mapping and Refinement of the Painted Turtle Genome (Chrysemys picta) Inform Amniote Genome Evolution and Challenge Turtle-Bird Chromosomal Conservation. *Genome Biol. Evol.* 7, 2038–2050. doi: 10.1093/gbe/evv119

Baker, R. J. (1967). Karyotypes of Bats of the Family Phyllostomidae and Their Taxonomic Implications. *Southwest. Nat.* 12, 407. doi: 10.2307/3669608

Baker, R. J., and Bleier, W. J. (1971). Karyotypes of bats of the subfamily Carolliinae (Mammalia; Phyllostomatidae) and their evolutionary implications. *Experientia* 27, 220–2. doi: 10.1007/bf02145907

Baker, R. J., Bull, J. J., and Mengden, G. A. (1971). Chromosomes ofElaphe subocularis (Reptilia: Serpentes), with the description of an in vivo technique for preparation of snake chromosomes. *Experientia* 27, 1228–1229. doi: 10.1007/BF02286946

Baker, R. J., and Lopez, G. (1970). Karyotypic Studies of the Insular Populations of Bats on Puerto Rico. *Caryologia* 23, 465–472. doi: 10.1080/00087114.1970.10796387

Baker, R. J., Mengden, G. A., and Bull, J. J. (1972). Karyotypic Studies of Thirty-Eight Species of North American Snakes. *Copeia* 1972, 257. doi: 10.2307/1442486

Baker, R. J., and Patton, J. L. (1967). Karyotypes and Karyotypic Variation of North American Vespertilionid Bats. *J. Mammal.* 48, 270. doi: 10.2307/1378031

Becak, M. L., Becak, W., and Denaro, L. (1972). Chromosome Polymorphism, Geographical Variation and Karyotypes in Sauria. *Caryologia* 25, 313–326. doi: 10.1080/00087114.1972.10796485

Becak, M. L., Becak, W., Roberts, F. L., Shoffner, R. N., Volpe, E. P., Benirschke, K., et al. (1971). *Chromosome Atlas: Fish, Amphibians, Reptiles and Birds: Volume 1*. Springer.

Becak, W. (1965). Constituicao cromossomica e mecanismo de determinaacao do sexo em ofidios sulamericanos. I. Aspectos cariotipicos. *Mem Inst Butantan* 32, 37–78.

Becak, W., Becak, M. L., and Nazareth, H. R. (1962). Karyotypic studies of two species of South American snakes (Boa constrictor amarali and Bothrops jararaca). *Cytogenetics* 1, 305–313.

Bedford, J. M. (1963). Morphological Changes in Rabbit Spermatozoa During Passage Through the Epididymis. *J. Reprod. Fertil.* 5, 169–177.

Beletti, M. E., Costa, L. F., and Viana, M. P. (2005). A comparison of morphometric characteristics of sperm from fertile Bos taurus and Bos indicus bulls in Brazil. *Anim. Reprod. Sci.* 85, 105–116. doi: 10.1016/j.anireprosci.2004.04.019

Bellastella, G., Cooper, T. G., Battaglia, M., Ströse, A., Torres, I., Hellenkemper, B., et al. (2010). Dimensions of human ejaculated spermatozoa in Papanicolaou-stained seminal and swim-up smears obtained from the Integrated Semen Analysis System (ISAS®). *Asian J. Androl.* 12, 871–879. doi: 10.1038/aja.2010.90

Benirschke, K., Low, R. J., and Ferm, V. H. (1969). “Cytogenetic Studies of Some Armadillos,” in *Comparative Mammalian Cytogenetics* (New York: Springer-Verlag).

Benirschke, K., Rüedi, D., Müller, H., Kumamoto, A. T., Wagner, K. L., and Downes, H. S. (1980). The unusual karyotype of the lesser kudu, Tragelaphus imberbis. *Cytogenet. Genome Res.* 26, 85–92.

Bertolotto, C. E. V., Rodrigues, M. T., and Yonenaga-Yassuda, Y. (2001). Banding patterns, multiple sex chromosome system and localization of telomeric (TTAGGG)n sequences by FISH on two species of Polychrus (Squamata, Polychrotidae). *Caryologia* 54, 217–226. doi: 10.1080/00087114.2001.10589229

Bickham, J. W. (1976). A Meiotic Analysis of Four Species of Turtles. *Genetica* 46, 193–198.

Biederman, B. M., Lin, C. C., Kuyt, E., and Drewien, R. C. (1982). Genome of the whooping crane. *J. Hered.* 73, 145–146.

Bishop, M. W. H., and Walton, A. (1960). “Spermatogenesis and the structure of mammalian spermatozoa,” in *Marshall’s Physiology of Reproduction* (London: Longman), 1–129.

Blengini, C. S., Juri, G. L., Chiaraviglio, M., Unates, D. R., and Naretto, S. (2020). Sperm Parameters in Pristidactylus achalensis (Squamata: Leiosauridae), a Lizard Endemic to the Highest Mountain Areas in Central Argentina. *Copeia* 108, 538–544. doi: 10.1643/CH-19-310

Blengini, C. S., Sergio, N., Gabriela, C., Giojalas, L. C., and Margarita, C. (2014). Variability in sperm form and function in the context of sperm competition risk in two Tupinambis lizards. *Ecol. Evol.* 4, 4080–4092. doi: 10.1002/ece3.1262

Bonnet, A., Thévenon, S., Claro, F., Gautier, M., and Hayes, H. (2001). Cytogenetic comparison between Vietnamese sika deer and cattle: R-banded karyotypes and FISH mapping. *Chromosome Res.* 9, 673–687. doi: 10.1023/a:1012908508488

Bonnet-Garnier, A., Claro, F., Thévenon, S., Gautier, M., and Hayes, H. (2003). Identification by R-banding and FISH of chromosome arms involved in Robertsonian translocations in several deer species. *Chromosome Res.* 11, 649–663. doi: 10.1023/a:1025981508867

Breed, W. G. (2004). The Spermatozoon of Eurasian Murine Rodents: Its Morphological Diversity and Evolution. *J. Morphol.* 261, 52–69. doi: 10.1002/jmor.10228

Breed, W. G., and Inns, R. W. (1985). Variation in sperm morphology of Australian Vespertilionidae and its possible phylogenetic significance. *Mammalia* 49, 105–108.

Brown, M. E., Converse, S. J., Chandler, J. N., Crosier, A. L., Lynch, W., Wildt, D. E., et al. (2015). Time within reproductive season, but not age or inbreeding coefficient, affects seminal and sperm quality in the whooping crane (Grus americana). *Reprod. Fertil. Dev.* 29, 294–306. doi: 10.1071/RD15251

Brum-Zorrilla, N., De Catalfo, G. H., Degiovanangelo, C., Wainberg, R. L., and De Fronza, T. G. (1990). Calomys laucha chromosome (Rodentia, Cricetidae) from Uruguay and Argentina. *Caryologia* 43, 65–77.

Brun, B., and Rumpler, Y. (1990). Seasonal Variation of Sperm Morphology in the Mayotte Brown Lemur (Eulemur fulvus mayottensis). *Folia Primatol* 55, 51–56. doi: 10.1159/000156499

Bury, R. B., Gress, F., and Gorman, G. C. (1970). Karyotypic Survey of Some Colubrid Snakes from Western North America. *Herpetologica* 26, 461–466.

Capanna, E., and Civitelli, M. V. (1964). Contributo Alla Conoscenza Della Cariologia dei Rinolofidi (Mammalia - Chiroptera). *Caryologia* 17, 361–371. doi: 10.1080/00087114.1964.10796132

Capanna, E., and Civitelli, M. V. (1965). Cariologia e Cariometria del Miniottero (Mammalia - Chiroptera). *Caryologia* 18, 541–546. doi: 10.1080/00087114.1965.10796188

Carvalho, N. D. M., Arias, F. J., Da Silva, F. A., Schneider, C. H., and Gross, M. C. (2015). Cytogenetic analyses of five amazon lizard species of the subfamilies Teiinae and Tupinambinae and review of karyotyped diversity the family Teiidae. *Comp. Cytogenet.* 9, 625–644. doi: 10.3897/CompCytogen.v9i4.5371

Carvalho, N. D. M., Carmo, E., Neves, R. O., Schneider, C. H., and Gross, M. C. (2016). Differential repetitive DNA composition in the centromeric region of chromosomes of Amazonian lizard species in the family Teiidae. *Comp. Cytogenet.* 10, 203–217. doi: 10.3897/CompCytogen.v10i2.7081

Castillo, A., Taddei, A. R., Schiavone, A., Fausto, A. M., Marzoni, M., and di Cossato, F. (2022). Semen qualitative parameters and spermatozoon ultrastructure of Phasianus colchicus mongolicus. *Ital. J. Anim. Sci.* 21, 1151–1159. doi: 10.1080/1828051X.2022.2098837

Castoe, T., De Koning, A. P. J., Hall, K., Card, D., Schield, D., Fujita, M., et al. (2013). The Burmese python genome reveals the molecular basis for extreme adaptation in snakes. *Proc. Natl. Acad. Sci. U. S. A.* 110. doi: 10.1073/pnas.1314475110

Cetica, P., Rahn, I. M., Merani, M., and Solari, A. (1997). Comparative Spermatology in Dasypodidae II (Chaetophractus vellerosus, Zaedyus pichiy, Euphractus sexcinctus,

Tolypeutes matacus, Dasypus septemcinctus & Dasypus novemcinctus). *Biocell* 21, 195–204.

Cetica, P., Sassaroli, J. C., Merani, M., and Solari, A. (1993). Comparative spermatology in Dasypodidae (Priodontes maximus, Chaetophractus villosus, Dasypus hybridus). *Biocell* 18, 89–103.

Chan, F. H. P., Cianfriglia, M., Echard, G., Fox, R. R., Gustavsson, I., Martin-DeLeon, P. A., et al. (1981). Standard karyotype of the laboratory rabbit, Oryctolagus cuniculus. *Cytogenet. Cell Genet.* 31, 240–248.

Chandley, A. C., Jones, R. C., Dott, H. M., Allen, W. R., and Short, R. V. (1974). Meiosis in interspecific equine hybrids. I. The male mule (Equus asinus X E. caballus) and hinny (E. caballus X E. asinus). *Cytogenet. Cell Genet.* 13, 330–341. doi: 10.1159/000130284

Chang, Y.-C., Yu, J.-F., Wang, T.-E., Chin, S.-C., Wei, Y.-S., Chen, T.-Y., et al. (2020). Investigation of epididymal proteins and general sperm membrane characteristics of Formosan pangolin (Manis pentadactyla pentadactyla). *BMC Zool.* 5, 15. doi: 10.1186/s40850-020-00064-4

Chen, L., Qiu, Q., Jiang, Y., Wang, K., Lin, Z., Li, Z., et al. (2019). Large-scale ruminant genome sequencing provides insights into their evolution and distinct traits. *Science* 364, eaav6202. doi: 10.1126/science.aav6202

Cho, K.-W., Youn, H.-Y., Watari, T., Tsujimoto, H., Hasegawa, A., and Satoh, H. (1997). A proposed nomenclature of the domestic cat karyotype. *Cytogenet. Genome Res.* 79, 71–78.

Christidis, L., Shaw, D. D., and Schodde, R. (1991). Chromosomal evolution in parrots, lorikeets and cockatoos (Aves: Psittaciformes). *Hereditas* 114, 47–56. doi: 10.1111/j.1601-5223.1991.tb00552.x

Ciccioli, M., and Poggio, L. (1993). Genome size in Calomys laucha and Calomys musculinus (Rodentia, Cricetidae). *Genet. Sel. Evol.* 25, 109–120.

Claro, F., Hayes, H., and Cribiu, E. P. (1993). The R- and G-Banded Karyotypes of the Sable Antelope (Hippotragus niger). *J. Hered.* 84, 481–484. doi: 10.1093/oxfordjournals.jhered.a111376

Claro, F., Hayes, H., and Cribiu, E. P. (1996). The Karyotype of the Addax and Its Comparison with Karyotypes of Other Species of Hippotraginae Antelopes. *Hereditas* 124, 223–227. doi: 10.1111/j.1601-5223.1996.00223.x

Coeti, R. Z., Antoniazzi, M. M., Sánchez, R., and Almeida-Santos, S. M. (2021). Sperm storage in coral snakes: A spermatozoa ultrastructural approach (Serpentes: Elapidae). *Zool. Anz.* 290, 49–57. doi: 10.1016/j.jcz.2020.11.005

Cohen, M. M., and Clark, H. F. (1967). The somatic chromosomes of five crocodilian species. *Cytogenet. Genome Res.* 6, 193–203.

Cole, C. J. (1970). Karyotypes and Evolution of the spznosus Group of Lizards in the Genus Sceloporus. *Am. Mus. Novit.* 2431, 1–48.

Cole, C. J. (1975). Karyotype and Systematic Status of the Sand Dune Lizard (Sceloporus graciosus arenicolous) of the American Southwest. *Herpetologica* 31, 288–293.

Cole, C. J. (1978). Karyotypes and Systematics of the Lizards in the variabilis, jalapae, and scalaris Species Groups of the Genus Sceloporus. *Am. Mus. Novit.* 2653, 1–13.

Cole, C. J., and Hardy, L. M. (2019). Karyotypes of Six Species of Colubrid Snakes from the Western Hemisphere, and the 140-Million-Year-Old Ancestral Karyotype of Serpentes. *Am. Mus. Novit.* 2019, 1–14. doi: 10.1206/3926.1

Colli, G. R., Teixeira, R. D., Scheltinga, D. M., Mesquita, D. O., Wiederhecker, H. C., and Báo, S. N. (2007). Comparative study of sperm ultrastructure of five species of teiid lizards (Teiidae, Squamata), and Cercosaura ocellata (Gymnophthalmidae, Squamata). *Tissue Cell* 39, 59–78. doi: 10.1016/j.tice.2006.12.001

Contreras, L. C., Torres-Mura, J. C., and Spotorno, A. E. (1990). The largest known chromosome number for a mammal, in a South American desert rodent. *Experientia* 46, 506–8. doi: 10.1007/bf01954248

Cribiu, E. P., Di Berardino, D., Di Meo, G. P., Eggen, A., Gallagher, D. S., Gustavsson, I., et al. (2001). International System for Chromosome Nomenclature of Domestic Bovids (ISCNDB 2000). *Cytogenet. Genome Res.* 92, 283–299. doi: 10.1159/000056917

Cucho, H., Alarcón, V., Ordóñez, C., Ampuero, E., Meza, A., and Soler, C. (2016). Puma (Puma concolor) epididymal sperm morphometry. *Asian J. Androl.* 18, 879–881. doi: 10.4103/1008-682X.187584

Cucho, H., Nina, G., Meza, A., Ccalta, R., Ordóñez, C., and Valverde, A. (2022). Morphometric subpopulations study of white-tailed deer (Odocoileus virginianus peruvianus) epididymal spermatozoa. *Agron. Mesoam.* 33. doi: 10.15517/am.v33i2.46938

Cui, K. H., Flaherty, S. P., Newble, C. D., Guerin, M. V., Napier, A. J., and Matthews, C. D. (1991). Collection and Analysis of Semen from the Common Marmoset (Callithrix jacchus). *J. Androl.* 12, 214–220.

Cummins, J. M., and Woodall, P. F. (1985). On mammalian sperm dimensions. *J. Reprod. Fertil.* 75, 153–175. doi: 10.1530/jrf.0.0750153

De Boer, L. E. M. (1974). Cytotaxonomy of the Platyrrhini (Primates). *Genen Phaenen* 17, 1–115.

De Boer, L. E. M. (1976). The somatic chromosome complements of 16 species of falconiformes (Aves) and the karyological relationships of the order. *Genetica* 46, 77–113. doi: 10.1007/BF00122519

de Lemos Pinto, M. M. P., Calixto, M. S., Souza, M. J., Araújo, A. P. T., Langguth, A., and Santos, N. (2012). Cytotaxonomy of the subgenus Artibeus (Phyllostomidae, Chiroptera) by characterization of species-specific markers. *Comp. Cytogenet.* 6, 17–28. doi: 10.3897/compcytogen.v6i1.1510

De Smet, W. H. O. (1978a). The Chomosomes of 23 Species of Snakes. *Acta Zool. Pathol. Antverp.* 70, 85–118.

De Smet, W. H. O. (1978b). The Chromosomes of 11 Species of Chelonia (Reptilia). *Acta Zool. Pathol. Antverp.* 70, 15–34.

De Smet, W. H. O. (1981). Description of the orcein stained karyotypes of 36 lizard species (Lacertilia, Reptilia) belonging to the families Teiidae, Scincidae, Lacertidae, Cordylidae and Varanidae (Autarchoglossa). *Acta Zool. Pathol. Antverp.* 76, 73–118.

de Sousa Barbosa, B., Silva, H. R., Tabosa, B. A., Nunes, T. P., Magalhaes, F. F., and Silva, L. M. (2019). Morphological and morphometric characterization of domestic cat epididymal sperm. *Reprod. Domest. Anim. Wiley* 54, 1630–1636. doi: 10.1111/rda.13572

de Souza, M. S., Barcellos, S. A., Dos Santos, M. da S., Gunski, R. J., Garnero, A. D. V., de Oliveira, E. H. C., et al. (2022). Microchromosome BAC-FISH Reveals Different Patterns of Genome Organization in Three Charadriiformes Species. *Animals* 12, 3052. doi: 10.3390/ani12213052

Derjusheva, S., Kurganova, A., Habermann, F., and Gaginskaya, E. (2004). High chromosome conservation detected by comparative chromosome painting in chicken, pigeon and passerine birds. *Chromosome Res.* 12, 715–723. doi: 10.1023/B:CHRO.0000045779.50641.00

Di-Nizo, C., Neves, C., Fernando Vilela, J., and Silva, M. J. D. J. (2014). New karyologycal data and cytotaxonomic considerations on small mammals from Santa Virgínia (Parque Estadual da Serra do Mar, Atlantic Forest, Brazil). *Comp. Cytogenet.* 8, 11–30. doi: 10.3897/compcytogen.v8i1.6430

dos Santos, P. A., Ribeiro, V. M. F., Alves, A. L. F., Silva, V. L. da, Nascimento, B. K. F., Satrapa, R. A., et al. (2020). Morphology, morphometry, and membrane integrity of epididymal spermatozoa of spotted pacas (Cuniculus paca, Linnaeus 1766). *Semina Ciênc Agrar*, 181–190.

dos Santos, R. M. L., Pellegrino, K. C. M., Rodrigues, M. T., and Yonenaga-Yassuda, Y. (2007). Banding patterns and chromosomal evolution in five species of neotropical Teiinae lizards (Squamata: Teiidae). *Genetica* 131, 231–240. doi: 10.1007/s10709-006-9133-2

dos Santos, R. M. L., Rodrigues, M. T., Yonenaga-Yassuda, Y., and Pellegrino, K. C. M. (2008). Differential staining and microchromosomal variation in karyotypes of four Brazilian species of Tupinambinae lizards (Squamata: Teiidae). *Genetica* 134, 261–266. doi: 10.1007/s10709-007-9233-7

Du, B., and Wang, D. (2006). C-values of seven marine mammal species determined by flow cytometry. *Zool. Sci* 23, 1017–20. doi: 10.2108/zsj.23.1017

du Plessis, L., and Soley, J. T. (2014). Light microscopic features and morphometry of sperm in the emu (Dromaius novaehollandiae). *Theriogenology* 81, 203–209. doi: 10.1016/j.theriogenology.2013.08.016

Dunnum, J. L., and Salazar-Bravo, J. (2010). Phylogeny, evolution, and systematics of the Galea musteloides complex (Rodentia: Caviidae). *J. Mammal.* 91, 243–259.

Dussex, N., Alberti, F., Heino, M. T., Olsen, R.-A., van der Valk, T., Ryman, N., et al. (2020). Moose genomes reveal past glacial demography and the origin of modern lineages. *BMC Genomics* 21, 854. doi: 10.1186/s12864-020-07208-3

Dutrillaux, B. (1979). Chromosomal evolution in Primates: Tentative phylogeny from Microcebus murinus (Prosimian) to man. *Hum. Genet.* 48, 251–314. doi: 10.1007/BF00272830

Falcione, C., Hernando, A., and Bressa, M. J. (2018). Comparative cytogenetic analysis in Erythrolamprus snakes (Serpentes: Dipsadidae) from Argentina. *Ann. Braz. Acad. Sci.* 90, 1417–1429.

Fan, H., Wu, Q., Wei, F., Yang, F., Ng, B. L., and Hu, Y. (2019). Chromosome-level genome assembly for giant panda provides novel insights into Carnivora chromosome evolution. *Genome Biol.* 20, 267. doi: 10.1186/s13059-019-1889-7

Fausto, A. M., Taddei, A. R., Batocco, F., Belardinelli, M. C., Carcupino, M., Schiavone, A., et al. (2023). Raptors bred in captivity: semen characteristics and assisted reproduction outcome in goshawk (Accipiter gentilis). *PeerJ* 22, 1–25. doi: 10.7717/peerj.15094

Fedyk, A., and Fedyk, S. (1970). Karyotypes of some species of vespertilionid bats from Poland. *Acta Theriol. (Warsz.)* 15, 295–302.

Feito, R., and Gallardo, M. (1982). Sperm Morphology of the Chilean Species of Ctenomys (Octodontidae). *J. Mammal.* 63, 658–661. doi: 10.2307/1380273

Fitri, W., Wahid, H., Rinalfi, P. T., Rosnina, Y., Raj, D., Donny, Y., et al. (2020). Digital massage for semen collection, evaluation and extension in Malaysian estuarine crocodile (Crocodylus porosus). *Asian Pac. J. Reprod.* 9, 104–108. doi: 10.4103/2305-0500.281080

Foote, A., and Bunskoek, P. (2022). The genome sequence of the killer whale, Orcinus orca (Linnaeus, 1758). *Wellcome Open Res.* 7, 250. doi: 10.12688/wellcomeopenres.18278.1

Forman, G. L. (1968). Comparative Gross Morphology of Spermatozoa of Two Families of North American Bats. *Univ. Kans. Sci. Bull.* 47, 901–928.

Forman, G. L., and Genoways, H. H. (1979). Sperm Morphology. *Biol. Bats New World Fam. Phyllostomatidae Part III*, 177–204.

Fredga, K. (1966). Chromosome studies in five species of South American rodents (Suborder Hystricomorpha). *Mamm. Chromosom. Newsl.* 20, 45.

Friend, G. F. (1936). The Sperms of the British Muridae. *J. Cell Sci.* 311, 419–443. doi: 10.1242/jcs.s2-78.311.419

Friesen, C. R., Kahrl, A. F., and Olsson, M. (2020). Sperm competition in squamate reptiles. *Philos. Trans. R. Soc. B Biol. Sci.* 375, 20200079. doi: 10.1098/rstb.2020.0079

Friesen, C. R., Uhrig, E. J., Bentz, E. J., Blakemore, L. A., and Mason, R. T. (2017). Correlated evolution of sexually selected traits: interspecific variation in ejaculates, sperm morphology, copulatory mate guarding, and body size in two sympatric species of garter snakes. *Behav. Ecol. Sociobiol.* 71, 180. doi: 10.1007/s00265-017-2414-1

Frykman, I. (1972). Chromosome studies of Mustela putorius in tissue culture. *Hereditas* 70, 59–67. doi: 10.1111/j.1601-5223.1972.tb00993.x

Furo, I. de O., Kretschmer, R., O'Brien, P. C., Pereira, J. C., Garnero, A. del V., Gunski, R. J., et al. (2020). Chromosomal Evolution in the Phylogenetic Context: A Remarkable Karyotype Reorganization in Neotropical Parrot Myiopsitta monachus (Psittacidae). *Front. Genet.* 11, 721.

Gage, M. J. G. (1998). Mammalian sperm morphometry. *Proc R Soc Lond B* 265, 97–103. doi: 10.1098/rspb.1998.0269

Gallardo, M. H., Mondaca, F. C., Ojeda, R. A., and Nelida, K. (2002). Morphological diversity in the sperms of Caviomorph rodents. *Mastozoologia Neotropical* 9, 159–170.

Gallardo, M. H., and Reise, D. (1992). Systematics of Aconaemys (Rodentia, Octodontidae). *J. Mammal.* 73, 779–788. doi: 10.2307/1382195

Gallina, S., Mandujano, S., Bello, J., Arevalo, H. F. L., and Weber, M. (2010). "White-Tailed Deer: Odocoileus virginianus (Zimmermann 1780)," in *Neotropical Cervidology* (Jaboticabal, SP, Brazil: Funep/IUCN), 101–118.

Gao, J., Zhang, Y., and Lin, W. (2000). Ultrastructure of the spermatozoon of Natrix piscater. *Chin. J. Appl. Environ. Biol.* 6, 344–348.

Garcia, J. P., and Pessôa, L. M. (2010). Karyotypic composition of bats from the Brazilian nuclear power plant, state of Rio de Janeiro. *Chiropt. Neotropical* 16, 1.

Geheb, K. L. (1992). A Survey of Karyotypic Variation in Eight Species of Phrynosomatid Lizards (Reptilia: Squamata). Beaumont, TX: Lamar University.

Genoways, H. H., Baker, R. J., Bickham, J. W., and Phillips, C. J. (2005). *Bats of Jamaica*. Texas Tech University.

Ghosh, A., Johnson, M. G., Osmanski, A. B., Louha, S., Bayona-Vásquez, N. J., Glenn, T. C., et al. (2020). A High-Quality Reference Genome Assembly of the Saltwater Crocodile, Crocodylus porosus, Reveals Patterns of Selection in Crocodylidae. *Genome Biol. Evol.* 12, 3635–3646. doi: 10.1093/gbe/evz269

Giovannotti, M., Trifonov, V. A., Paoletti, A., Kichigin, I. G., O'Brien, P. C. M., Kasai, F., et al. (2017). New insights into sex chromosome evolution in anole lizards (Reptilia, Dactyloidae). *Chromosoma* 126, 245–260. doi: 10.1007/s00412-016-0585-6

Giugliano, L. G., Teixeira, R. D., Colli, G. R., and Bao, S. N. (2002). Ultrastructure of Spermatozoa of the Lizard Ameiva ameiva, With Considerations on Polymorphism Within the Family Teiidae (Squamata). *J. Morphol.* 253, 264–271. doi: 10.1002/jmor.10002

Gomes, A. J. B., Nagamachi, C. Y., Rodrigues, L. R. R., Benathar, T. C. M., Ribas, T. F. A., O'Brien, P. C. M., et al. (2016). Chromosomal phylogeny of Vampyressine bats (Chiroptera, Phyllostomidae) with description of two new sex chromosome systems. *BMC Evol. Biol.* 16, 119. doi: 10.1186/s12862-016-0689-x

Goodpasture, C., Seluja, G., and Gee, G. (1992). Karyotype and identification of sex in two endangered crane species. in *North American Crane Workshop Proceedings* , 219–224.

Gorman, G. (1973). "The chromosomes of the Reptilia, a cytotaxonomic interpretation," in *Cytotaxonomy and Vertebrate Evolution* (New York: Academic Press), 349–424.

Gorman, G., Atkins, L., and Holzinger, T. (1967). New karyotypic data on 15 genera of lizards in the family Iguanidae, with a discussion of taxonomic and cytological implications. *Cytogenetics* 6, 286–299.

Gorman, G. C. (1970). Chromosomes and the Systematics of the Family Teiidae (Sauria, Reptilia). *Copeia* 1970, 230. doi: 10.2307/1441645

Gosch, B., and Fischer, K. (1989). Seasonal changes of testis volume and sperm quality in adult fallow deer (Dama dama) and their relationship to the antler cycle. *J. Reprod. Fertil.* 85, 7–17. doi: 10.1530/jrf.0.0850007

Gould, K. G., and Martin, D. E. (1978). Comparative Morphology of Primate Spermatozoa using Scannlng Electron Microscopy. II. Families Cercopithecidae, Lorisidae, Lemuridae. *J. Hum. Evol.* 7, 637–640. doi: 10.1016/S0047-2484(78)80048-7

Greenbaum, I. F., Baker, R. J., and Wilson, D. E. (1975). Evolutionary implications of the karyotypes of the stenodermine genera Ardops, Ariteus, Phyllops, and Ectophylla. *Bull. South. Calif. Acad. Sci.* 74, 156–159.

Gregory, T. R. (2019). Animal Genome Size Database. Available at: www.genomesize.com

Gribbins, K. M., Gist, D. H., and Congdon, J. D. (2003). Cytological evaluation of spermatogenesis and organization of the germinal epithelium in the male slider turtle, Trachemys scripta. *J. Morphol.* 255, 337–346. doi: 10.1002/jmor.10069

Gribbins, K. M., Matchett, C. L., DelBello, K. A., Rheubert, J., Villagrán-SantaCruz, M., Granados-González, G., et al. (2014). The ultrastructure of spermatid development during spermiogenesis within the rosebelly lizard, Sceloporus variabilis (Reptilia, Squamata, Phrynosomatidae). *J. Morphol.* 275, 258–268. doi: 10.1002/jmor.20212

Gribbins, K. M., Touzinsky, K. F., Siegel, D. S., Venable, K. J., Hester, G. L., and Elsey, R. M. (2011). Ultrastructure of the Spermatozoon of the American Alligator, Alligator mississippiensis (Reptilia: Alligatoridae). *J. Morphol.* 272, 1281–1289. doi: 10.1002/jmor.10984

Guo, C., and Ma, H. (1997). Studies of the karyotype,C-banding pattern and Ag-NORs of Elaphe schrenckii anomal. *Hered. Beijing* 19, 20–22.

Gustavsson, I. (1964). The chromosomes of the dog. *Hereditas* 51, 187–189. doi: 10.1111/j.1601-5223.1964.tb01926.x

Hamilton, A. E., and Buettner-Janusch, J. (1977). Chromosomes of Lemuriformes III. The Genus Lemur: Karyotypes of Species, Subspecies, and Hybrids. *Ann N Acad Sci* 293, 125–159.

Hammar, B. (1966). The Karyotypes of Nine Birds. *Hereditas* 55, 367–385.

Hammar, B. (1970). The karyotypes of thirty-one birds. *Hereditas* 65, 29–58.

Hansen, K. M. (1973a). Q-band karyotype of the goat (Capra hircus) and the relation between goat and bovine Q-bands. *Hereditas* 75, 119–130. doi: 10.1111/j.1601-5223.1973.tb01148.x

Hansen, K. M. (1973b). The karyotype of the domestic sheep (Ovis aries) identified by quinacrine mustard staining and fluorescence microscopy. *Hereditas* 75, 233–240. doi: 10.1111/j.1601-5223.1973.tb01164.x

Hao, S., Pan, L., Fang, Z., and Zhang, Y. (2015). Comparative Studies on Sperm Ultrastructure of Three Gecko Species, Gekko japonicus, Gekko chinensis and Hemidactylus bowrigii (Reptilia, Squamata, Gekkonidae). *Asian Herpetol. Res.* 6, 189–198.

Harnden, D. G., and Klinger, H. P. (1985). *An International System for Human Cytogenetic Nomenclature (1985)*. New York: Karger.

Hayata, I., Sonta, S.-I., Itoh, M., and Kondo, N. (1971). Notes on the karyotypes of some prosimians, Lemur mongoz, Lemur catta, Nycticebus coucang and Galago crassicaudatus. *Jpn. J. Genet.* 46, 61–64.

Healy, J. M., and Jamieson, B. G. M. (1992). Ultrastructure of the Spermatozoon of the Tuatara (Sphenodon punctatus) and its Relevance to the Relationships of the Sphenodontida. *Phil Trans R Soc Lond B* 335, 192–205. doi: 10.1098/rstb.1992.0018

Heath, E., Schaeffer, N., Meritt, D. A., and Jeyendran, R. S. (1987). Rouleaux formation by spermatozoa in the naked-tail armadillo, Cabassous unicinctus. *J. Reprod. Fertil.* 79, 153–158. doi: 10.1530/jrf.0.0790153

Herzog, S., Herzog, A., Höhn, H., Matern, B., and Hecht, W. (1992). Chromosome polymorphism in Ateles geoffroyi (Cebidae; Primates; Mammalia). *Theor. Appl. Genet.* 84, 986–989. doi: 10.1007/BF00227414

Hess, R. A., Thurston, R. J., and Gist, G. H. (1991). Ultrastructure of the Turtle Spermatozoon. *Anat. Rec.* 229, 473–481. doi: 10.1002/ar.1092290406

Hirth, H. F. (1960). The Spermatozoa of some North American Bats and Rodents. *J. Morphol.* 106, 77–83. doi: 10.1002/jmor.1051060103

Hoffmann, R., Faust, R., Hoffmann-fezer, G., and Weinand, U. (1974). Karyotypen von Kagu (Rhynochetos jubatus), Klunkerkranich (Bugeranus carunculatus) und Schuhschnabel (Balaeniceps rex). *Zool Gart. N F Jena* 6, 349–356.

Hosli, V. P., and Lang, E. M. (1970). Die Chromosomen des Davidshirsches (Elaphurus davidianus). *Arch. Suisses Médecine Vét.* 112, 395–396.

Houck, M. L., Kingswood, S. C., and Kumamoto, A. T. (2000). Comparative cytogenetics of tapirs, genus Tapirus (Perissodactyla, Tapiridae). *Cytogenet. Genome Res.* 89, 110–115. doi: 10.1159/000015587

Houck, M. L., Kumamoto, A. T., Gallagher, D. S., and Benirschke, K. (2001). Comparative cytogenetics of the African elephant (Loxodonta africana) and Asiatic elephant (Elephas maximus). *Cytogenet. Cell Genet.* 93, 249–252. doi: 10.1159/000056992

Hsu, T. C., and Arrighi, F. E. (1971). Distribution of Constitutive Heterochromatin in Mammalian Chromosomes. *Chromosoma* 34, 243–253.

Hsu, T. C., Rearden, H. H., and Luquette, G. F. (1963). Karyological Studies of Nine Species of Felidae. *Am. Nat.* 97, 225–234.

Huang, C. C., and Gans, C. (1971). The chromosomes of 14 species of amphisbaenians (Amphisbaenia, Reptilia). *Cytogenetics* 10, 10–22. doi: 10.1159/000130122

Huang, L., Nesterenko, A., Nie, W., Wang, J., Su, W., Graphodatsky, A. S., et al. (2008). Karyotype evolution of giraffes (Giraffa camelopardalis) revealed by cross-species chromosome painting with Chinese muntjac (Muntiacus reevesi) and human (Homo sapiens) paints. *Cytogenet. Genome Res.* 122, 132–138.

Hungerford, D. A., Chandra, H. S., and Snyder, R. L. (1967). Somatic Chromosomes of a Black Rhinoceros (Diceros bicornis Gray 1821). *Am. Nat.* 101, 357–358.

Immler, S., Saint-Jalme, M., Lesobre, L., Sorci, G., Roman, Y., and Birkhead, T. R. (2007). The evolution of sperm morphometry in pheasants. *J. Evol. Biol.* 20, 1008–1014. doi: 10.1111/j.1420-9101.2007.01302.x

Itoh, M., IKEUCHI, T., SHIMBA, H., MORI, M., Sasaki, M., and Makino, S. (1969). A comparative karyotype study in fourteen species of birds. *Jpn. J. Genet.* 44, 163–170.

Ivanov, V. G., and Bogdanov, O. P. (1975). Karyotype of the lizard Eumeces taeniolatus. *Tsitologiia* 17, 861–863.

Jainudeen, M. R., Eisenberg, J. F., and Jayasinghe, J. B. (1971). Semen on the Ceylon elephant, Elephas maximus. *J. Reprod. Fertil.* 24, 213–217. doi: 10.1530/jrf.0.0240213

Jamieson, B. G. M., and Koehler, L. (1994). The ultrastructure of the spermatozoon of the northern water snake, Nerodia sipedon (Colubridae, Serpentes), with phylogenetic considerations. *Can. J. Zool.* 72, 1648–1652.

Jamieson, B. G. M., and Scheltinga, D. M. (1994). The Ultrastructure of Spermatozoa of the Australian Skinks, Ctenotus Taeniolatus, Carlia Pectoralis and Tiliqua Scincoides Scincoides (Scincidae, Reptilia). *Mem. Qld. Mus.* 37, 181–193.

Jarrell, G. H., and Arnason, U. (1981). Banded karyotypes of a belukha whale, Delphinapterus leucas. *Hereditas* 95, 37–41. doi: 10.1111/j.1601-5223.1981.tb01326.x

Johnson, D. D. P., and Briskie, J. V. (1999). Sperm Competition and Sperm Length in Shorebirds. *The Condor* 101, 848–854. doi: 10.2307/1370074

Jones, J. K., and Baker, R. J. (1980). Chiroderma improvisum. *Mamm. Species* 134, 1–2.

Joseph, S., O'Connor, R., Al Mutery, A., Watson, M., Larkin, D., and Griffin, D. (2018). Chromosome Level Genome Assembly and Comparative Genomics between Three Falcon Species Reveals an Unusual Pattern of Genome Organisation. *Diversity* 10, 113. doi: 10.3390/d10040113

Kahrl, A. F., Johnson, M. A., and Cox, R. M. (2019). Rapid evolution of testis size relative to sperm morphology suggests that post-copulatory selection targets sperm number in Anolis lizards. *J. Evol. Biol.* 32, 302–309. doi: 10.1111/jeb.13414

Kasahara, S., Yonenaga-Yassuda, Y., Schincariol, R. A., and L'Abbate, M. (1983). Chromosome mechanisms of sex determination, G- and C-band patterns and nucleolus organizer regions in Tropidurus torquatus (Sauria, Iguanidae). *Genetica* 60, 151–156. doi: 10.1007/BF00127501

Kawagoshi, T., Nishida, C., and Matsuda, Y. (2012). The origin and differentiation process of X and Y chromosomes of the black marsh turtle (Siebenrockiella crassicollis, Geoemydidae, Testudines). *Chromosome Res.* 20, 95–110. doi: 10.1007/s10577-011-9267-7

Khongcharoensuk, H., Tanomtong, A., Patawang, I., Supanuam, P., Sornnok, S., and Pinthong, K. (2017). Karyotype and Idiogram of the Axis Deer (Axia axis, Cervidae) by Conventional Staining, GTG-, High-Resolution GTG-, and Ag-NOR-Banding Techniques. *Cytologia (Tokyo)* 82, 91–98. doi: 10.1508/cytologia.82.91

Kihlberg, C.-G. (1969). The Mitotic and Meiotic Chromosomes of the Muskrat (Ondatra zibethica L.). *Acta Vet Scand* 10, 181–192.

Kilpatrick, C. W., and Zimmerman, E. G. (1973). Karyology of North American Natricine Snakes (Family Colubridae) of the Genera Natrix and Regina. *Can. J. Genet. Cytol.* 15, 355–361.

Kim, H.-H., and Lee, J.-H. (2011). Fine Structure of the Sperm in the Myotis daubentonii ussuriensis. *Appl. Microsc.* 41.

Kimsakulvech, S., and Suttiyotin, P. (2020). Ultrastructural characteristics of black marsh turtle spermatozoa obtained by electroejaculation. *Anat. Histol. Embryol.* 49, 842–847. doi: 10.1111/ahe.12592

King, M., Honeycutt, R., and Contreras, N. (1986). Chromosomal repatterning in crocodiles: C, G and N-banding and the in situ hybridization of 18S and 26S rRNA cistrons. *Genetica* 70, 191–201. doi: 10.1007/BF00122186

Kingswood, S. C., Kumamoto, A. T., Charter, S. J., and Houck, M. L. (2000). Chromosomes of the antelope genus Kobus (Artiodactyla, Bovidae): karyotypic divergence by centric fusion rearrangements. *Cytogenet. Cell Genet.* 91, 128–133.

Kita, S., Yoshioka, M., Kashiwagi, M., Ogawa, S., and Tobayama, T. (2001). Comparative external morphology of cetacean spermatozoa. *Fish. Sci.* 67, 482–492. doi: 10.1046/j.1444-2906.2001.00284.x

Klinger, H. P. (1963). The Somatic Chromosomes of Some Primates (Tupaia Glis, Nycticebus Coucang, Tarsius Bancanus, Cercocebus Aterrimus, Symphalangus Syndactylus). *Cytogenetics* 2, 140–151. doi: 10.1159/000129775

Koepfli, K.-P., Tamazian, G., Wildt, D., Dobrynin, P., Kim, C., Frandsen, P. B., et al. (2019). Whole Genome Sequencing and Re-sequencing of the Sable Antelope (Hippotragus niger): A Resource for Monitoring Diversity in ex Situ and in Situ Populations. *G3 GenesGenomesGenetics* 9, 1785–1793. doi: 10.1534/g3.119.400084

Konvalina, J., Stanley, J., Trauth, S., and Plummer, M. (2020). No Sperm Morphometric Differences between Two Populations of Diamond-backed Watersnakes (Nerodia rhombifer) with Varying Resource Availability. *Copeia* 108, 376. doi: 10.1643/CG-17-692

Kostmann, A., Augstenová, B., Frynta, D., Kratochvíl, L., and Rovatsos, M. (2021). Cytogenetically Elusive Sex Chromosomes in Scincoidean Lizards. *Int. J. Mol. Sci.* 22, 8670. doi: 10.3390/ijms22168670

Koulischer, L., Tyskens, J., and Mortelmans, J. (1972). Mammalian cytogenetics. VII. The chromosomes of Cervus canadensis, Elaphurus davidianus, Cervus nippon (Temminck) and Pudu pudu. *Acta Zool. Pathol. Antverp.* 56, 25–30.

Kretschmer, R., de Souza, M. S., Barcellos, S. A., Degrandi, T. M., Pereira, J. C., O'Brien, P. C. M., et al. (2020). Novel insights into chromosome evolution of Charadriiformes: extensive genomic reshuffling in the wattled jacana (Jacana jacana, Charadriiformes, Jacanidae). *Genet. Mol. Biol.* 43, e20190236. doi: 10.1590/1678-4685-GMB-2019-0236

Krishan, A., and Shoffner, R. N. (1966). Sex chromosomes in the domestic fowl (Gallus domesticus), turkey (Meleagris gallopavo) and the Chinese pheasant (Phasianus colchicus). *Cytogenet. Genome Res.* 5, 53–63.

Ledesma, M. A., Ledesma, M. A., Freitas, T. R. O. de, Silva, J. da, Silva, F. R. da, and Gunski, R. J. (2003). Descripción cariotípica de Spheniscus magellanicus (Spheniscidae). *El Hornero* 18, 61–64.

Liming, S., Yingying, Y., and Xingsheng, D. (1980). Comparative cytogenetic studies on the red muntjac, Chinese muntjac, and their F1 hybrids. *Cytogenet. Cell Genet.* 26, 22–27.

Lin, L.-K., Ma, G.-C., Chen, T.-H., Lin, W.-H., Lee, D.-J., Wen, P.-Y., et al. (2013). Genomic analyses of the Formosan harvest mouse (Micromys minutus) and comparisons to the brown Norway rat (Rattus norvegicus) and the house mouse (Mus musculus). *Zoology* 116, 307–315. doi: 10.1016/j.zool.2013.07.001

Lioi, M. B., Scarfi, M. R., and Di Berardino, D. (1994). The RBA-banded karyotype of the fallow deer (Dama dama L.). *Cytogenet. Cell Genet.* 67, 75–80. doi: 10.1159/000133803

Lipshutz, S. E., Torneo, S. J., and Rosvall, K. A. (2023). How Female-Female Competition Affects Male-Male Competition: Insights into Postcopulatory Sexual Selection from Socially Polyandrous Species. *Am. Nat.* 201, 460–471. doi: 10.1086/722799

Liu, B., Jin, G. L., Zhao, S. H., Yu, M., Xiong, T. A., Peng, Z. Z., et al. (2002). Preparation and analysis of spermatocyte meiotic pachytene bivalents of pigs for gene mapping. *Cell Res.* 12, 401–405. doi: 10.1038/sj.cr.7290142

Liu, Y., Liu, S., Zhang, N., Chen, D., Que, P., Liu, N., et al. (2019). Genome Assembly of the Common Pheasant Phasianus colchicus: A Model for Speciation and Ecological Genomics. *Genome Biol. Evol.* 11, 3326–3331. doi: 10.1093/gbe/evz249

London, E. W., Roca, A. L., Novakofski, J. E., and Mateus-Pinilla, N. E. (2022). A De Novo Chromosome-Level Genome Assembly of the White-Tailed Deer, Odocoileus Virginianus. *J. Hered.* 113, 479–489. doi: 10.1093/jhered/esac022

Luther, I., Maree, L., Kotze, A., Hildebrandt, T., Göritz, F., Hermes, R., et al. (2020). Sperm motility, kinematics, morphometry and morphology over two seasons in free-ranging African elephants (Loxodonta africana). *Reprod. Fertil. Dev.* 32, 425–438. doi: 10.1071/RD19182

Madeddu, M., Berlinguer, F., Ledda, M., Leoni, G. G., Satta, V., Succu, S., et al. (2009). Ejaculate collection efficiency and post-thaw semen quality in wild-caught Griffon

vultures from the Sardinian population. *Reprod. Biol. Endocrinol.* 7, 18. doi: 10.1186/1477-7827-7-18

Mafunda, P. S., Maree, L., Kotze, A., and van der Horst, G. (2017). Sperm structure and sperm motility of the African and Rockhopper penguins with special reference to multiple axonemes of the flagellum. *Theriogenology* 99, 1–9. doi: 10.1016/j.theriogenology.2017.05.009

Majhi, R. K., Kumar, A., Yadav, M., Kumar, P., Maity, A., Giri, S. C., et al. (2016). Light and electron microscopic study of mature spermatozoa from White Pekin duck (Anas platyrhynchos): an ultrastructural and molecular analysis. *Andrology* 4, 232–244. doi: 10.1111/andr.12130

Makinen, A. (1985). The standard karyotype of the silver fox (Vulpes fulvus Desm.). *Hereditas* 103, 171–176.

Manier, M. K., Welch, G., Van Nispen, C., Bakst, M. R., and Long, J. (2019). Low-mobility sperm phenotype in the domestic turkey: Impact on sperm morphometry and early embryonic death. *Reprod. Domest. Anim.* 54, 613–621. doi: 10.1111/rda.13403

Maroto-Morales, A., Ramón, M., García-Álvarez, O., Montoro, V., Soler, A. J., Fernández-Santos, M. R., et al. (2015). Sperm head phenotype and male fertility in ram semen. *Theriogenology* 84, 1536–1541. doi: 10.1016/j.theriogenology.2015.07.038

Masuda, R., Noro, M., Kurose, N., Nishida-Umehara, C., Takechi, H., Yamazaki, T., et al. (1998). Genetic characteristics of endangered Japanese golden eagles (Aquila chrysaetos japonica) based on mitochondrial DNA D-loop sequences and karyotypes. *Zoo Biol.* 17, 111–121. doi: 10.1002/(SICI)1098-2361(1998)17:2<111::AID-ZOO6>3.0.CO;2-C

Millán de la Blanca, M. G., Martínez-Nevado, E., Castaño, C., García, J., Bernal, B., Toledano-Díaz, A., et al. (2021). Sperm Cryopreservation in American Flamingo (Phoenicopterus Ruber): Influence of Cryoprotectants and Seminal Plasma Removal. *Anim. Basel* 11, 203. doi: 10.3390/ani11010203

Miller, D. L., Steer, E. L., Decker, S. J., and Robeck, T. (2002). Ultrastructure of the Spermatozoa from Three Odontocetes: a Killer Whale (Orcinus orca), a Pacific White-Sided Dolphin (Lagenorhynchus obliquidens) and a Beluga (Delphinapterus leucas). *Anat. Histol. Embryol.* 31, 158–168. doi: 10.1046/j.1439-0264.2002.00385.x

Misra, M., and Srivastava, M. D. L. (1976). Somatic chromosomes of Bubulcus ibis (L.) (Cattle-Egret): A case of reciprocal translocation. *Genetica* 46, 155–160. doi: 10.1007/BF00121031

Mogoe, T., Fukui, Y., Ishikawa, H., and Ohsumi, S. (1998). Morphological Observations of Frozen-Thawed Spermatozoa of Southern Minke Whales (Balaenoptera acutorostrata). *J. Reprod. Dev.* 44, 95–100. doi: 10.1262/jrd.44.95

Mudry, M. D., Nieves, M., and Steinberg, E. R. (2015). "Cytogenetics of Howler Monkeys," in *Howler Monkeys: Adaptive Radiation, Systematics, and Morphology* , eds. M. K. Martin, A. G. Paul, C.-O. Liliana, U. Bernardo, and Y. Dionisios (Springer, New York), 85–105.

Murata, M., and Murakami, M. (2014). Two Distinct mtDNA Lineages among Captive African Penguins in Japan. *J. Vet. Med. Sci.* 76, 559–563. doi: 10.1292/jvms.13-0377

Nanda, I., Karl, E., Griffin, D. K., Schartl, M., and Schmid, M. (2007). Chromosome repatterning in three representative parrots (Psittaciformes) inferred from comparative chromosome painting. *Cytogenet. Genome Res.* 117, 43–53. doi: 10.1159/000103164

NCBI (2023). Genome search portal, National Center for Biotechnology Information. Available at: https://www.ncbi.nlm.nih.gov/datasets/genome/

Newton, W. D., and Trauth, S. E. (1992). Ultrastructure of the Spermatozoon of the Lizard Cnemidophorus sexlineatus (Sauria: Teiidae). *Herpetologica* 48, 330–343.

Nie, W., O'Brien, P. C. M., Fu, B., Wang, J., Su, W., He, K., et al. (2015). Multidirectional chromosome painting substantiates the occurrence of extensive genomic reshuffling within Accipitriformes. *BMC Evol. Biol.* 15, 205. doi: 10.1186/s12862-015-0484-0

Nie, W., Wang, J., Su, W., Wang, Y., and Yang, F. (2009). Chromosomal rearrangements underlying karyotype differences between Chinese pangolin (Manis pentadactyla) and Malayan pangolin (Manis javanica) revealed by chromosome painting. *Chromosome Res.* 17, 321–329. doi: 10.1007/s10577-009-9027-0

Nishida, C., Ishijima, J., Kosaka, A., Tanabe, H., Habermann, F. A., Griffin, D. K., et al. (2008). Characterization of chromosome structures of Falconinae (Falconidae, Falconiformes, Aves) by chromosome painting and delineation of chromosome rearrangements during their differentiation. *Chromosome Res.* 16, 171–181. doi: 10.1007/s10577-007-1210-6

Nishida-Umehara, C., Tsuda, Y., Ishijima, J., Ando, J., Fujiwara, A., Matsuda, Y., et al. (2007). The molecular basis of chromosome orthologies and sex chromosomal differentiation in palaeognathous birds. *Chromosome Res.* 15, 721–734. doi: 10.1007/s10577-007-1157-7

Nishida-Umehara, C., and Yoshida, M. C. (1994). The karyotype of nine golden eagles, Aquila chrysaetos. *Chrom Inf. Serv* 56, 22–24.

Norris, T. B., Rickards, G. K., and Daugherty, C. H. (2004). Chromosomes of tuatara, Sphenodon, a chromosome heteromorphism and an archaic reptilian karyotype. *Cytogenet. Genome Res.* 105, 93–99. doi: 10.1159/000078014

O'Brien, J. K., Oehler, D. A., Malowski, S. P., and Roth, T. L. (1999). Semen collection, characterization, and cryopreservation in a Magellanic penguin (Spheniscus magellanicus). *Zoo Biol.* 18, 199–214. doi: 10.1002/(SICI)1098-2361(1999)18:3<199::AID-ZOO4>3.0.CO;2-#

O'Brien, S. J., Graphodatsky, A. S., and Perelman, P. L. (2020). *Atlas of Mammalian Chromosomes*. 2nd ed. New York: John Wiley & Sons.

Ogawa, A., Murata, K., and Mizuno, S. (1998). The location of Z- and W-linked marker genes and sequence on the homomorphic sex chromosomes of the ostrich and the emu. *Proc. Natl. Acad. Sci.* 95, 4415–4418. doi: 10.1073/pnas.95.8.4415

Ohno, S. (1967). *Sex Chromosomes and Sex-linked Genes*. Berlin: Springer-Verlag.

Okura, N., Shiraishi, S., and Uchida, T. (1984). Karyotypes of the Japanese Harvest Mouse (Micromys minutus japonicus) from Fukuoka and the Tsushima Islands. *J. Fac. Agric. Kyushu Univ.* 28, 177–183. doi: 10.5109/23787

Oliveira, V. C. S., Altmanová, M., Viana, P. F., Ezaz, T., Bertollo, L. A. C., Ráb, P., et al. (2021). Revisiting the Karyotypes of Alligators and Caimans (Crocodylia, Alligatoridae) after a Half-Century Delay: Bridging the Gap in the Chromosomal Evolution of Reptiles. *Cells* 10, 1397. doi: 10.3390/cells10061397

O'Meally, D., Miller, H., Patel, H. R., Marshall Graves, J. A., and Ezaz, T. (2009). The First Cytogenetic Map of the Tuatara, Sphenodon punctatus. *Cytogenet. Genome Res.* 127, 213–223. doi: 10.1159/000300099

Patterson, M. C., Stover, J., Westrom, W. K., and Schaffer, N. S. (1985). Comparative Dimensional Analysis of Spermatozoa of Seven Species of Cervidae. *J. Zoo Anim. Med.* 16, 144–146. doi: 10.2307/20094772

Peng, C., Wu, D.-D., Ren, J.-L., Peng, Z.-L., Ma, Z., Wu, W., et al. (2023). Large-scale snake genome analyses provide insights into vertebrate development. *Cell* 186, 2959-2976.e22. doi: 10.1016/j.cell.2023.05.030

Pennock, L. A., Tinkle, D. W., and Shaw, M. W. (1969). Minute Y chromosome in the lizard genus Uta (family Iguanidae). *Cytogenetics* 8, 9–19. doi: 10.1159/000130018

Pereira, H. R. J., Santiloni, V., Rosa, P., Mota, L., and Jorge, W. (2009). The karyotype of Cabassous unicinctus (Dasypodidae, Xenar-thra). *Caryologia* 62, 24–29. doi: 10.1080/00087114.2004.10589662

Piccinni, E., and Stella, M. (1970). Some avian karyograms. *Caryologia* 23, 189–202.

Pigozzi, M. I. (2011). Diverse stages of sex-chromosome differentiation in tinamid birds: evidence from crossover analysis in Eudromia elegans and Crypturellus tataupa. *Genetica* 139, 771–777. doi: 10.1007/s10709-011-9581-1

Pinna-Senn, E., Di Tada, I., and Lisanti, J. (1987). Polymorphism Of The Microchromosomes And The Nucleolar Organizer Region In Pristidactylus achalensis (Sauria: Iguanidae). *Herpetologica* 43, 120–127.

Rao, L., Turlapati, R., Patel, M., Panda, B., Tosh, D., Mangalipalli, S., et al. (2009). Cytogenetic characterization and fluorescence in situ hybridization of (GATA)10 repeats on

established primary cell cultures from Indian water snake (Natrix piscator) and Indian mugger (Crocodylus palustris) embryos. *Cytogenet. Genome Res.* 127, 287–296. doi: 10.1159/000304046

Ray-Chaudhuri, R., Sharma, T., and Ray-Chaudhuri, S. P. (1969). A Comparative Study of the Chromosomes of Birds. *Chromosoma* 26, 148–168.

Rheubert, J., Messak, J. A., Siegel, D. S., Gribbins, K. M., Trauth, S. E., and Sever, D. M. (2017). Inter- and intraspecific variation in sperm morphology of Sceloporus consobrinus and Sceloporus undulatus (Squamata: Phrynosomatidae). *Biol. J. Linn. Soc.* 121, 355–364. doi: 10.1093/biolinnean/blw043

Richard, F., Lombard, M., and Dutrillaux, B. (2003). Reconstruction of the ancestral karyotype of eutherian mammals. *Chromosome Res.* 11, 605–618.

Richer, C. L., Power, M. M., Klunder, L. R., McFEELY, R. A., and Kent, M. G. (1990). Standard karyotype of the domestic horse (Equus caballus). *Hereditas* 112, 289–293. doi: 10.1111/j.1601-5223.1990.tb00069.x

Robson, S., Rouse, G., and Pettigrew, J. (1997). Sperm Ultrastructure of Tarsius bancanus (Tarsiidae, Primates): Implications for Primate Phylogeny and the Use of Sperm in Systematics. *Acta Zool* 78, 269–278. doi: 10.1111/j.1463-6395.1997.tb01011.x

Roopnarine, N. H., Gupta, S. K., du Plessis, L., and Aire, T. A. (2020). Sperm structure of the cattle egret (Bubulcus ibis). *Anat. Histol. Embryol.* 49, 814–819. doi: 10.1111/ahe.12586

Rossi, L. F., de la Sancha, N. U., Luaces, J. P., Estevez, D. Y., and Merani, M. S. (2018). Morphological description and comparison of sperm from eighteen species of cricetid rodents. *J. Mammal.* 99, 1398–1404. doi: 10.1093/jmammal/gyy146

Sandfoss, M. R., Reichling, S., and Roberts, B. M. (2023). Sperm morphology and forward motility are indicators of reproductive success and are not age- or condition-dependent in a captive breeding population of endangered snake. *PLOS ONE* 18, e0282845. doi: 10.1371/journal.pone.0282845

Santiago-Moreno, J., Esteso, M. C., Pradiee, J., Castaño, C., Toledano-Díaz, A., O'Brien, E., et al. (2016). Giant panda (Ailuropoda melanoleuca) sperm morphometry and function after repeated freezing and thawing. *Andrologia* 48, 470–474. doi: 10.1111/and.12468

Santos, N., Fagundes, V., Yonenaga-Yassuda, Y., and De Souza, M. J. (2001). Comparative karyology of Brazilian vampire bats Desmodus rotundus and Diphylla ecaudata (Phyllostomidae, Chiroptera): banding patterns, base-specific fluorochromes and FISH of ribosomal genes. *Hereditas* 134, 189–194. doi: 10.1111/j.1601-5223.2001.00189.x

Santos, N., and Souza, M. J. D. (1998). Characterization of the constitutive heterochromatin of Carollia perspicillata (Phyllostomidae, Chiroptera) using the base-specific fluorochromes,

CMA3 (GC) and DAPI (AT). *Caryologia* 51, 51–60. doi: 10.1080/00087114.1998.10589119

Saravia, F., Núñez-Martínez, I., Morán, J. M., Soler, C., Muriel, A., Rodríguez-Martínez, H., et al. (2007). Differences in boar sperm head shape and dimensions recorded by computer-assisted sperm morphometry are not related to chromatin integrity. *Theriogenology* 68, 196–203. doi: 10.1016/j.theriogenology.2007.04.052

Sasaki, M., Ikeuchi, T., and Makino, S. (1968). A feather pulp culture technique for avian chromosomes, with notes on the chromosomes of the peafowl and the ostrich. *Experientia* 24, 1292–1293. doi: 10.1007/BF02146680

Scheltinga, D. M., Jamieson, B. G., Trauth, S. E., and McAllister, C. T. (2000). Morphology of the spermatozoa of the iguanian lizards Uta stansburiana and Urosaurus ornatus (Squamata, Phrynosomatidae). *J. Submicrosc. Cytol. Pathol.* 32, 261–271.

Schmid, M., Enderle, E., Schindler, D., and Schempp, W. (1989). Chromosome banding and DNA replication patterns in bird karyotypes. *Cytogenet. Genome Res.* 52, 139–146.

Schmid, M., and Steinlein, C. (2017). The Hypermethylated Regions in Avian Chromosomes. *Cytogenet. Genome Res.* 151, 216–227. doi: 10.1159/000464268

Schwenk, K., Sessions, S. K., and Seale, D. M. P. (1982). Karyotypes of the Basiliscine Lizards Corytophanes cristatus and Corytophanes hernandesii, with Comments on the Relationship between Chromosomal and Morphological Evolution in Lizards. *Herpetologica* 38, 493–501.

SDZWA (2023). San Diego Zoo Wildlife Alliance (SDZWA) Sperm Atlas. Available at: https://library.sandiegozoo.org/sperm-atlas/

Sebestova, H., Vozdova, M., Kubickova, S., Cernohorska, H., Kotrba, R., and Rubes, J. (2016). Effect of species-specific differences in chromosome morphology on chromatin compaction and the frequency and distribution of RAD51 and MLH1 foci in two bovid species: cattle (Bos taurus) and the common eland (Taurotragus oryx). *Chromosoma* 125, 137–149. doi: 10.1007/s00412-015-0533-x

Serafim, H., Peccinini-Seale, D. M., and Batistic, R. F. (2007). Estudo cariotípico de duas espécies brasileiras do gênero Micrurus (Ophidia: Elapidae). *Biota Neotropica* 7, 75–80. doi: 10.1590/S1676-06032007000100010

Shibaike, Y., Takahashi, Y., Arikura, I., Iiizumi, R., Kitakawa, S., Sakai, M., et al. (2009). Chromosome evolution in the lizard genus Gekko (Gekkonidae, Squamata, Reptilia) in the East Asian islands. *Cytogenet. Genome Res.* 127, 182–190. doi: 10.1159/000303334

Shibusawa, M., Nishida-Umehara, C., Masabanda, J., Griffin, D. K., Isobe, T., and Matsuda, Y. (2002). Chromosome rearrangements between chicken and guinea fowl defined by

comparative chromosome painting and FISH mapping of DNA clones. *Cytogenet. Genome Res.* 98, 225–230. doi: 10.1159/000069813

Shindo, J., Abe, T., Yamaguchi, T., and Kobayashi, K. (2000). A Scanning Electron Microscope Study of Humboldt Penguin (*Spheniscus humboldti*) Spermatozoa. *Jpn. J. Zoo Wildl. Med.* 5, 105–109. doi: 10.5686/jjzwm.5.105

Silva, M. J. da, Cipriano, F. M. G., Vieira, A. P. de A., Cândido, M. R. dos S., Pinheiro, T. G., and Silva, E. L. da (2020). Cytogenetic characterization of Ameivula ocellifera (Spix, 1825) (Squamata, Teiidae) from the brazilian northeast. *Biosci. J.* 36, 1018–1023. doi: 10.14393/BJ-v36n3a2020-49880

Silva, H. V. R., Nunes, T. G. P., Ribeiro, L. R., Freitas, L. A. de, de Oliveira, M. F., Assis Neto, A. C. de, et al. (2019). Morphology, morphometry, ultrastructure, and mitochondrial activity of jaguar (Panthera onca) sperm. *Anim. Reprod. Sci.* 203, 84–93. doi: 10.1016/j.anireprosci.2019.02.011

Silveira, J. A. (2009). Testicular histology and characterization of the stages of the seminiferous epithelial cycle of Hemidactylus mabouia (Moreau de Jonnès, 1818) (Reptilia, Squamata, Sauria, Gekkonidae). Universidade Federal de Viçosa.

Singchat, W., Sillapaprayoon, S., Muangmai, N., Baicharoen, S., Indananda, C., Duengkae, P., et al. (2020). Do sex chromosomes of snakes, monitor lizards, and iguanian lizards result from multiple fission of an “ancestral amniote super-sex chromosome”? *Chromosome Res.* 28, 209–228. doi: 10.1007/s10577-020-09631-4

Singh, L. (1972). Evolution of karyotypes in snakes. *Chromosoma* 38, 185–236. doi: 10.1007/BF00326193

Singh, L., Sharma, T., and Ray-Chaudhuri, S. P. (1968). Chromosomes and the classification of the snakes of the family Boidae. *Cytogenet. Genome Res.* 7, 161–168.

Snow, J. L., Jones, J. K., Jr., and Webster, Wm. D. (1980). Centurio senex. *Mamm. Species* 138, 1–3. doi: 10.2307/3503871

Sotero-Caio, C. G., Pieczarka, J. C., Nagamachi, C. Y., Gomes, A. J. B., Lira, T. C., O’Brien, P. C. M., et al. (2011). Chromosomal Homologies among Vampire Bats Revealed by Chromosome Painting (Phyllostomidae, Chiroptera). *Cytogenet. Genome Res.* 132, 156–164. doi: 10.1159/000321574

Sotero-Caio, C. G., Volleth, M., Hoffmann, F. G., Scott, L., Wichman, H. A., Yang, F., et al. (2015). Integration of molecular cytogenetics, dated molecular phylogeny, and model-based predictions to understand the extreme chromosome reorganization in the Neotropical genus Tonatia (Chiroptera: Phyllostomidae). *BMC Evol. Biol.* 15, 220. doi: 10.1186/s12862-015-0494-y

Spangenberg, V., Arakelyan, M., Galoyan, E., Matveevsky, S., Petrosyan, R., Bogdanov, Y., et al. (2017). Reticulate Evolution of the Rock Lizards: Meiotic Chromosome Dynamics and Spermatogenesis in Diploid and Triploid Males of the Genus Darevskia. *Genes* 8, 149–164. doi: 10.3390/genes8060149

Spotorno, A. E., Walker, L. I., Contreras, L. C., Torres, J. C., Fernando-Donoso, R., Berrios, M. S., et al. (1995). Chromosome divergence of Octodon lunatus and Abrocoma bennetti and the origins of Octodontoidea (Rodentia: Histricognathi). *Rev. Chil. Hist. Nat.* 68, 227–239.

Steinberg, E. R., Sestelo, A. J., Ceballos, M. B., Wagner, V., Palermo, A. M., and Mudry, M. (2019). Sperm Morphology in Neotropical Primates. *Animals* 9, 839–854. doi: 10.3390/ani9100839

Stelzer, G., Schmidt, V., Sobiraj, A., and Krautwald-Junghanns, M.-E. (2009). Spermatozoa Characteristics in Six Psittacine Species Using Light Microscopy. *Reprod. Domest. Anim.* 44, 894–899. doi: 10.1111/j.1439-0531.2008.01113.x

Suryamohan, K., Krishnankutty, S. P., Guillory, J., Jevit, M., Schröder, M. S., Wu, M., et al. (2020). The Indian cobra reference genome and transcriptome enables comprehensive identification of venom toxins. *Nat. Genet.* 52, 106–117. doi: 10.1038/s41588-019-0559-8

Takagi, N., and Makino, S. (1966). A Revised Study on the Chromosomes of three Species of Birds. *Caryologia* 19, 443–455. doi: 10.1080/00087114.1966.10796235

Takagi, N., and Sasaki, M. (1974). A Phylogenetic Study of Bird Karyotypes. *Chromosoma* 46, 91–120.

Tamsitt, J. R., and Nagorsen, D. (1982). Anoura cultrata. *Mamm. Species* 179, 1–5.

Tavares, J. R., Sousa, T. P. D., Silva, J. M. D., Venere, P. C., and Faria, K. D. C. (2015). Cytogenetics and DNA barcoding of the Round-eared bats, Tonatia (Chiroptera: Phyllostomidae): a new karyotype for Tonatia bidens. *Zool. Curitiba* 32, 371–379. doi: 10.1590/S1984-46702015000500006

Tavares-Bastos, L., Teixeira, R. D., Colli, G. R., and Bao, S. N. (2002). Polymorphism in the sperm ultrastructure among four species of lizards in the genus Tupinambis (Squamata: Teiidae). *Acta Zool. Stockh.* 83, 297–307. doi: 10.1046/j.1463-6395.2002.00119.x

Teixeira, R. D., Colli, G. R., and Bao, S. N. (1999a). The ultrastructure of the spermatozoa of the lizard Micrablepharus maximiliani (Squamata, Gymnophthalmidae), with considerations on the use of sperm ultrastructure characters in phylogenetic reconstruction. *Acta Zool.* 80, 47–59. doi: 10.1046/j.1463-6395.1999.20010.x

Teixeira, R. D., Colli, G. R., and Báo, S. N. (1999b). The ultrastructure of the spermatozoa of the worm lizard Amphisbaena alba (Squamata, Amphisbaenidae) and the phylogenetic relationships of amphisbaenians. *Can. J. Zool.* 77, 1254–1264. doi: 10.1139/z99-089

Teixeira, R. D., Scheltinga, D. M., Trauth, S. E., Colli, G. R., and Báo, S. N. (2002). A comparative ultrastructural study of spermatozoa of the teiid lizards Cnemidophorus gularis gularis, Cnemidophorus ocellifer, and Kentropyx altamazonica (Reptilia, Squamata, Teiidae). *Tissue Cell* 34, 135–142. doi: 10.1016/s0040-8166(02)00021-6

Thurston, R. J., Hess, R. A., Hughes, B. L., and Froman, D. P. (1982). Ultrastructure of the Guinea Fowl (Numidia meleagris) Spermatozoon. *Poult. Sci.* 61, 1738–1743. doi: 10.3382/ps.0611738

Tourmente, M., Cardozo, G., Bertona, M., Guidobaldi, A., Giojalas, L., and Chiaraviglio, M. (2006). The ultrastructure of the spermatozoa of Boa constrictor occidentalis, with considerations on its mating system and sperm competition theories. *Acta Zool.* 87, 25–32. doi: 10.1111/j.1463-6395.2006.00217.x

Tourmente, M., Giojalas, L., and Chiaraviglio, M. (2008). Sperm ultrastructure of Bothrops alternatus and Bothrops diporus (Viperidae, Serpentes), and its possible relation to the reproductive features of the species. *Zoomorphology* 127, 241–248. doi: 10.1007/s00435-008-0067-3

Tourmente, M., Gomendio, M., Roldan, E. R. S., Giojalas, L. C., and Chiaraviglio, M. (2009). Sperm Competition and Reproductive Mode Influence Sperm Dimensions and Structure among Snakes. *Evolution* 63, 2513–2524. doi: 10.1111/j.1558-5646.2009.00739.x

Tsuda, Y., Nishida-Umehara, C., Ishijima, J., Yamada, K., and Matsuda, Y. (2007). Comparison of the Z and W sex chromosomal architectures in elegant crested tinamou (Eudromia elegans) and ostrich (Struthio camelus) and the process of sex chromosome differentiation in palaeognathous birds. *Chromosoma* 116, 159–173. doi: 10.1007/s00412-006-0088-y

van der Horst, G., Curry, P. T., Kitchin, R. M., Burgess, W., Thorne, E. T., Kwiatkowski, D., et al. (1991). Quantitative Light and Scanning Electron Microscopy of Ferret Sperm. *Mol. Reprod. Dev.* 30, 232–240. doi: 10.1002/mrd.1080300311

Velhankar, D. P., Hukeri, V. B., Deshpande, B. R., and Sane, C. R. (1973). Biometry of the genitalia and the spermatozoa of a male giraffe. *Indian Vet. J.* 50, 789–792.

Ventura, K., Silva, M. J. de J., Fagundes, V., Pardini, R., and Yonenaga-Yassuda, Y. (2004). An undescribed karyotype for Thaptomys (2n= 50) and the mechanism of differentiation from Thaptomys nigrita (2n= 52) evidenced by FISH and Ag-NORs. *Caryologia* 57, 89–97.

Vernon, G. G., and Woolley, D. M. (1999). Three-Dimensional Motion of Avian Spermatozoa. *Cell Motil. Cytoskeleton* 42, 149–161. doi: 10.1002/(SICI)1097-0169(1999)42:2<149::AID-CM6>3.0.CO;2-0

Veronese, L. B., de Freitas, T. R. O., and Krause, L. (2003). Cytogenetic studies of four Brazilian species of teiid lizards (Squamata, Teiidae). *Caryologia* 56, 107–114. doi: 10.1080/00087114.2003.10589313

Viana, P. F., Ezaz, T., De Bello Cioffi, M., Liehr, T., Al-Rikabi, A., Goll, L. G., et al. (2020). Landscape of snake' sex chromosomes evolution spanning 85 MYR reveals ancestry of sequences despite distinct evolutionary trajectories. *Sci. Rep.* 10, 12499. doi: 10.1038/s41598-020-69349-5

Viana, P. F., Ribeiro, L. B., Souza, G. M., Chalkidis, H. D. M., Gross, M. C., and Feldberg, E. (2016). Is the Karyotype of Neotropical Boid Snakes Really Conserved? Cytotaxonomy, Chromosomal Rearrangements and Karyotype Organization in the Boidae Family. *PLoS ONE* 11, e0160274. doi: 10.1371/journal.pone.0160274

Vieira, G. H. C., Colli, G. R., and Báo, S. N. (2004). The ultrastructure of the spermatozoon of the lizard Iguana iguana (Reptilia, Squamata, Iguanidae) and the variability of sperm morphology among iguanian lizards. *J. Anat.* 204, 451–464. doi: 10.1111/j.0021-8782.2004.00300.x

Vieira, G. H. C., Colli, G. R., and Bao, S. N. (2005). Phylogenetic relationships of corytophanid lizards (Iguania, Squamata, Reptilia) based on partitioned and total evidence analyses of sperm morphology, gross morphology, and DNA data. *Zool. Scr.* 34, 605–625. doi: 10.1111/j.1463-6409.2005.00208.x

Vieira, G. H. C., Cunha, L. D., Scheltinga, D. M., Glaw, F., Colli, G. R., and Báo, S. N. (2007). Sperm ultrastructure of hoplocercid and oplurid lizards (Sauropsida, Squamata, Iguania) and the phylogeny of Iguania. *J. Zool. Syst. Evol. Res.* 45, 230–241. doi: 10.1111/j.1439-0469.2007.00406.x

Villaverde-Morcillo, S., Esteso, M. C., Castano, C., Toledano Diaz, A. T., Lopez-Sebastian, A., Campo, J. L., et al. (2015a). Influence of Staining Method on the Values of Avian Sperm Head Morphometric Variables. *Reprod. Domest. Anim.* 50, 750–755. doi: 10.1111/rda.12574

Villaverde-Morcillo, S., García-Sánchez, R., Castaño, C., Rodríguez, E., Gonzalez, F., Esteso, M., et al. (2015b). Characterization of Natural Ejaculates and Sperm Cryopreservation in a Golden Eagle (Aquila Chrysaetus). *J. Zoo Wildl. Med.* 46, 335–338. doi: 10.1638/2013-0293R1.1

Villaverde-Morcillo, S., Soler, A. J., Esteso, M. C., Miñano-Berna, A., Gonzalez, F., and Santiago-Moreno, J. (2017). Immature and mature sperm morphometry in fresh and

frozen-thawed falcon ejaculates. *Theriogenology* 98, 94–100. doi: 10.1016/j.theriogenology.2017.04.051

Walen, K. H., and Madin, S. H. (1965). Comparative Chromosome Analyses of the Bottlenosed Dolphin (Tursiops truncatus) and the Pilot Whale (Globicephala scammonii). *Am. Nat.* 99, 349–354. doi: 10.1086/282376

Walker, L. I., Soto, M. A., and Spotorno, A. E. (2014). Similarities and differences among the chromosomes of the wild guinea pig Cavia tschudii and the domestic guinea pig Cavia porcellus (Rodentia, Caviidae). *CompCytogen* 8, 153–167. doi: 10.3897/CompCytogen.v8i2.7509

Wang, B., Wang, Z., Zhou, J., Liu, W., Lin, Z., Zhang, C., et al. (2020a). The Draft Genome of Red Lechwe, Kobus leche leche. *Front. Genet.* 11, 582638. doi: 10.3389/fgene.2020.582638

Wang, S.-S., Lu, Y.-Y., Yao, C.-H., Qi, S., Cheng, J.-X., Lu, S.-X., et al. (2020b). Ultrastructure of Spermatozoa of Elaphe schrenckii (Reptilia, Squamata). *Russ. J. Herpetol.* 27. doi: 10.30906/1026-2296-2020-27-3-149-155

Warr, A., Affara, N., Aken, B., Beiki, H., Bickhart, D. M., Billis, K., et al. (2020). An improved pig reference genome sequence to enable pig genetics and genomics research. *GigaScience* 9, giaa051. doi: 10.1093/gigascience/giaa051

Willis, K. B., Willig, M. R., and Jones, J. K. (1990). Vampyrodes caraccioli. *Mamm. Species* 359, 1. doi: 10.2307/3504287

Wittayarat, M., Pukazhenthi, B. S., Tipkantha, W., Techakumphu, M., Srisuwatanasagul, S., and Panyaboriban, S. (2021). CRISP protein expression in semen of the endangered Malayan tapir (Tapirus indicus). *Theriogenology* 172, 106–115. doi: 10.1016/j.theriogenology.2021.06.005

Woolley, D. M. (1995). The structure of the spermatozoon of the Japanese quail Coturnix coturnix L., var. japonica. *Acta Zool* 76, 45–50.

Wurster, D. H., and Benirschke, K. (1967). Chromosome Studies in Some Deer, the Springbok, and the Pronghorn, with Notes on Placentation in Deer. *Cytologia (Tokyo)* 32, 273–285.

Wurster, D. H., and Benirschke, K. (1970). Indian muntjac, Muntiacus muntjak: a deer with a low diploid chromosome number. *Science* 168, 1364–1366.

Wurster, D. H., Benirschke, K., and Noelke, H. (1968). Unusually large sex chromosomes in the sitatunga (Tragelaphus spekei) and the blackbuck (Antilope cervicapra). *Chromosoma* 23, 317–323. doi: 10.1007/BF02451003

Xing, X., Ai, C., Wang, T., Li, Y., Liu, H., Hu, P., et al. (2023). The First High-quality Reference Genome of Sika Deer Provides Insights into High-tannin Adaptation. *Genomics Proteomics Bioinformatics* 21, 203–215. doi: 10.1016/j.gpb.2022.05.008

Yadav, J. S., Pachlag, S., Burra, M. R., and Yadav, A. S. (1995). Karyotypic analysis of three species of Phasianidae (Galliformes; Aves). *Cytobios* 81, 119–127.

Yang, Y., Gao, Z., and Zhao, E. (1989). Karyotypic studies of Sphenomorphus indicus (Scincidae) and Takydromus septentrionalis (Lacertidae). *Chin. Herpetol. Res.* 2, 55–59.

Yaseen, A. E. (1998). Karyological Studies on Some Bird Species. *Cytologia (Tokyo)* 63, 155–169. doi: 10.1508/cytologia.63.155

Yim, H.-S., Cho, Y. S., Guang, X., Kang, S. G., Jeong, J.-Y., Cha, S.-S., et al. (2014). Minke whale genome and aquatic adaptation in cetaceans. *Nat. Genet.* 46, 88–92. doi: 10.1038/ng.2835

Yonenaga, Y., Frota-Pessoa, O., and Lewis, K. R. (1969). Karyotypes of Seven Species of Brazilian Bats. *Caryologia* 22, 63–79. doi: 10.1080/00087114.1969.10796325

Yonenaga-Yassuda, Y., and Rodrigues, M. T. (1999). Supernumerary chromosome variation, heteromorphic sex chromosomes and banding patterns in microteiid lizards of the genus Micrablepharus (Squamata, Gymnophthalmidae). *Chromosome Res.* 7, 21–29.

Yu, F.-J., Zeng, C.-J., Zhang, Y., Wang, C.-D., Xiong, T.-Y., Fang, S.-G., et al. (2015). Establishment and Cryopreservation of a Giant Panda Skeletal Muscle-Derived Cell Line. *Biopreservation Biobanking* 13, 195–199. doi: 10.1089/bio.2014.0073

Zhang, Y., Ying, X., and Ji, X. (2005). Ultrastructure of the Spermatozoon of the Northern Grass Lizard (Takydromus septentrionalis) with Comments on the Variability of Sperm Morphology Among Lizard Taxa. *Zool. Res.* 26, 518–526.

Zimmerman, E. G., and Kilpatrick, C. W. (1973). Karyology of North American crotaline snakes (family Viperidae) of the genera Agkistrodon, Sistrurus, and Crotalus. *Can. J. Genet. Cytol.* 15, 389–395.
