# Supplementary material for: Karyotype depends on sperm head morphology in some amniote groups
Source: Front Genet. 2024 Jun 6;15:1396530. doi: 10.3389/fgene.2024.1396530 (PMC11186999; doi:10.3389/fgene.2024.1396530)
Supplement: Supplementary file 2 [file DataSheet1.pdf]

Supplementary Material for E. M. Kramer et al. (2024)

Karyotype depends on sperm head morphology in some amniote groups

Contents:

I. Species excluded from the analysis.
II. Use of TimeTree.
III. OLS regression details.
IV. PGLS regression details.
V. References.
VI. Plots of the Phylogenetic Trees.
VII. Table S1. Database summary.

I. Species excluded from the analysis.

As discussed in the main text, we excluded any species or phylogenetic group with a sperm head that didn't match the stereotyped morphologies of spatulate or vermiform heads (Fig. 1). We also excluded species or groups where the nucleus didn't occupy at least 50% of the midline length of the sperm head (in the literature, such cases are often described as having "giant" acrosomes). This allowed us to use the head morphology as a proxy for nuclear morphology.

Mammalia. Our analysis of mammals was limited to sperm head shapes that may be classified as spatulate, paddle-shaped, bullet-shaped, oval, or ellipsoidal. This excludes most rodents (order Rodentia), as these typically have an elongated and curved acrosome that forms a distal "hook" or more complicated shapes (Breed, 2005). Our database does include 15 rodent species with an oval or spatulate head. Second, we exclude all marsupials (infraclass Marsupialia), as marsupial sperm heads often have complex shapes with pronounced concavities or folds (Roldan et al., 1992). Third, we exclude isolated genera of primates that have evolved a fold or swelling along the distal edge of the acrosome [e.g. (Gould and Martin, 1978)]. Fourth, we exclude all of order Eulipotyphla (moles, shrews, etc., previously classed as Insectivora), as the acrosome can be larger than the nucleus itself and/or adopt complex shapes [e.g. (Castellani-

Ceresa et al., 1980; Parapanov et al., 2009)]. Fifth is the bat genus Noctilio, with giant acrosomes (Forman et al., 1989).

One mammal species in the database that might qualify as an ambiguous “edge” case is *Manis pentadactyla* (pangolin), as its sperm head is longer and more cylindrical than typical (Chang et al., 2020). However, the apex of the head is still flattened, and the ultrastructure of the nucleus and acrosome is otherwise typical of spatulate mammals, so it was included in our analysis.

Sauropsids. Most bird and non-avian reptile species have an approximately cylindrical, conical, or vermiform head shape, with a relatively small acrosomal complex at the distal end. From the sauropsids, we only excluded order Passeriformes (passerine birds), as these have a more complex helical or spindle-shaped head with an acrosome that can be longer than the nucleus, and a membranous structure that winds in a helix around the head (Støstad et al., 2018).

II. Use of TimeTree

Phylogenetic trees for Mammalia and Sauropsida were generated using TimeTree version 5 (Kumar et al., 2022). To generate a complete phylogeny, TimeTree automatically made congeneric substitutions for *Giraffa camelopardalis*, *Miniopterus schreibersii*, *Eulemur fulvus*, *Jacana jacana*, *Phoenicopterus ruber*, and *Gyps fulvus*. Similarly, we substituted *Teius teyou* for *Teius oculatus*, *Eptesicus fuscus miradorensis* for *Eptesicus fuscus*, and *Crotallus molossus molossus* for *Crotallus molossus* in the input list for TimeTree. In no case did our database include a second species in the same genus that might confound the use of a substitution. TimeTree produces a phylogenic tree in Newick format, which was subsequently imported into R. Plots of the phylogenetic trees are appended to this Supplement.

III. OLS regression details.

L_{min}. We conducted a simultaneous ordinary least squares (OLS) regression of L_{min} on genome size C, chromosome dispersity K, and chromosome number n:

$$\log_{10}(L_{min}) = \delta + \alpha \log_{10}(C) + \beta \log_{10}(K) + \gamma \log_{10}(n)$$

Param.	value	p < .05
α	0.124 ± 0.160	N
β	-0.630 ± 0.097	Y
γ	-0.568 ± 0.106	Y
δ	1.294 ± 0.171	Y

N = 65 entries and $R^2 = 0.715$.

IV. PGLS regression details.

Gross morphology. We tested the importance of sperm head morphology by introducing the parameter s (s = 0 for mammals, s = 1 for sauropsids). The PGLS regression is for $\log_{10}(n) = \alpha + \beta s$ and $\log_{10}(K) = \alpha + \beta s$. Reported p-values are from 2-sided t-tests with the null hypothesis $\beta = 0$.

	α	β	λ	p < .05	N
N vs. s	1.392 ± 0.121	-.042 ± 0.143	.604	N	193
K vs. s	0.740 ± 0.290	0.338 ± 0.340	.942	N	193

Karyotype regressions on sperm head and genome size. The following PGLS linear models are, in all cases, $\log_{10}(y) = \log_{10}(A) + \beta*\log_{10}(x)$, the double-log transform of $y = Ax^{\beta}$. Reported p-values are from 2-sided t-tests with the null hypothesis $\beta = 0$.

We tested the dependence of K and n on C for the combined set of sauropsids and mammals.

y vs. x	$\log_{10}(A)$	β	λ	p < .05	N
n vs. C	1.281 ± 0.084	0.227 ± 0.140	.555	N	142
K vs. C	1.046 ± 0.159	-0.128 ± 0.169	.931	N	142

For Fig. 4, panels A and D, N = 103 (all mammal species in database). The two rows with head length L do not correspond to figures in the main text. They are provided to demonstrate the qualitative similarity of area and length as independent variables. In the main text we prefer to use area for spatulate heads since it is more directly related to nuclear shape changes during spermiogenesis, as described in the Discussion section.

Panel	y vs. x	$\log_{10}(A)$	β	λ	p < .05	N
4A	n vs. A	1.088 ± 0.102	0.226 ± 0.070	.329	Y	103
4D	K vs. A	0.710 ± 0.128	0.022 ± 0.075	.880	N	103
	n vs. L	1.011 ± 0.127	0.452 ± 0.146	.286	Y	103
	K vs. L	0.729 ± 0.160	0.015 ± 0.164	0.882	N	103

For Fig. 4, panels B, C, E, and F, N =87 (we have genome size for 87/103 = 84% of mammal entries). The two rows showing n vs. A and K vs. A with N = 87 do not correspond to figures in the main text. They are provided for comparison with the N = 103 data set above.

Panel	y vs. x	$\log_{10}(A)$	β	λ	p < .05	N
	n vs. A	1.101 ± 0.106	0.214 ± 0.074	.268	Y	87
4B	n vs. C	1.095 ± 0.115	0.565 ± 0.214	.214	Y	87
4C	n vs. A/C	1.234 ± 0.081	0.189 ± 0.084	.300	Y	87
	K vs. A	0.734 ± 0.132	0.002 ± 0.079	.851	N	87
4E	K vs. C	0.832 ± 0.143	-0.182 ± 0.231	.862	N	87
4F	K vs A/C	0.714 ± 0.104	0.028 ± 0.087	.848	N	87

For Fig 5, panels A and D, N = 90 species (all sauropsid species in database).

Panel	y vs. x	Log10(A)	β	λ	p < .05	N
5A	n vs. L	1.313 ± 0.109	0.024 ± 0.077	.963	N	90
5D	K vs. L	0.559 ± 0.224	0.427 ± 0.171	.708	Y	90

For Fig. 5, panels B, C, E, and F, N = 55 species (we have genome size for 55/90 = 61% of sauropsid entries). The two rows showing n vs. L and K vs. L with N = 55 do not correspond to

figures in the main text. They are provided for comparison with the N = 90 data set above. The exponent β of the K vs. L regression (highlighted with an asterisk below) is not significant in the smaller set.

Panel		Log10(A)	β	λ	p < .05	N
	n vs. L	1.236 ± 0.117	0.087 ± 0.085	.988	N	55
5B	N vs. C	1.357 ± 0.059	-0.040 ± 0.093	.978	N	55
5C	N vs. L/C	1.279 ± 0.080	0.072 ± 0.070	.980	N	55
	K vs. L	0.875 ± 0.272	0.178 ± 0.202*	.922	N*	55
5E	K vs. C	1.104 ± 0.139	-0.026 ± 0.234	.949	N	55
5F	K vs. L/C	0.700 ± 0.157	0.445 ± 0.168	.305	Y	55

V. References.

Breed, W. G. (2005). Evolution of the Spermatozoon in Muroid Rodents. *J. Morphol.* 265, 271–290.

Castellani-Ceresa, L., Cotelli, F., Lora-Lamia, C., and Berruti, G. (1980). The Spermatozoon of Talpa europaea with Particular Reference to the Acrosome Region. *J. Ultrastruct. Res.* 72, 112–118.

Chang, Y.-C., Yu, J.-F., Wang, T.-E., Chin, S.-C., Wei, Y.-S., Chen, T.-Y., et al. (2020). Investigation of epididymal proteins and general sperm membrane characteristics of Formosan pangolin (Manis pentadactyla pentadactyla). *BMC Zool.* 5, 15. doi: 10.1186/s40850-020-00064-4

Forman, G. L., Smith, J. D., and Hood, C. S. (1989). Exceptional Size and Unusual Morphology of Spermatozoa in Noctilio albiventris (Noctilionidae). *J. Mammal.* 70, 179–184. doi: 10.2307/1381684

Gould, K. G., and Martin, D. E. (1978). Comparative Morphology of Primate Spermatozoa using Scannlng Electron Microscopy. II. Families Cercopithecidae, Lorisidae, Lemuridae. *J. Hum. Evol.* 7, 637–640. doi: 10.1016/S0047-2484(78)80048-7

Kumar, S., Suleski, M., Craig, J. M., Kasprowicz, A. E., Sanderford, M., Li, M., et al. (2022). TimeTree 5: An Expanded Resource for Species Divergence Times. *Mol. Biol. Evol.* 39, msac174. doi: 10.1093/molbev/msac174

Parapanov, R. N., Nusslé, S., Crausaz, M., Senn, A., Haussas, J., and Vogel, P. (2009). Testis size, sperm characteristics and testosterone concentrations in four species of shrews (Mammalia, Soricidae). *Anim. Reprod. Sci.* 114, 269–278. doi: 10.1016/j.anireprosci.2008.09.013

Roldan, E. R. S., Gomendio, M., and Vitullo, A. D. (1992). The Evolution of Eutherian Spermatozoa and Underlying Selective Forces: Female Selection and Sperm Competition. *Biol. Rev.* 67, 551–593.

Støstad, H. N., Johnsen, A., Lifjeld, J. T., and Rowe, M. (2018). Sperm head morphology is associated with sperm swimming speed. *Evolution* 72, 1918–1932. doi: 10.1111/evo.13555

VI. Plots of phylogenetic trees.

Fig S1. Phylogenetic tree of mammal species in the database. Tip-to-root 99.2 MY. Generated using TimeTree v 5.0 (Kumar et al., 2022).

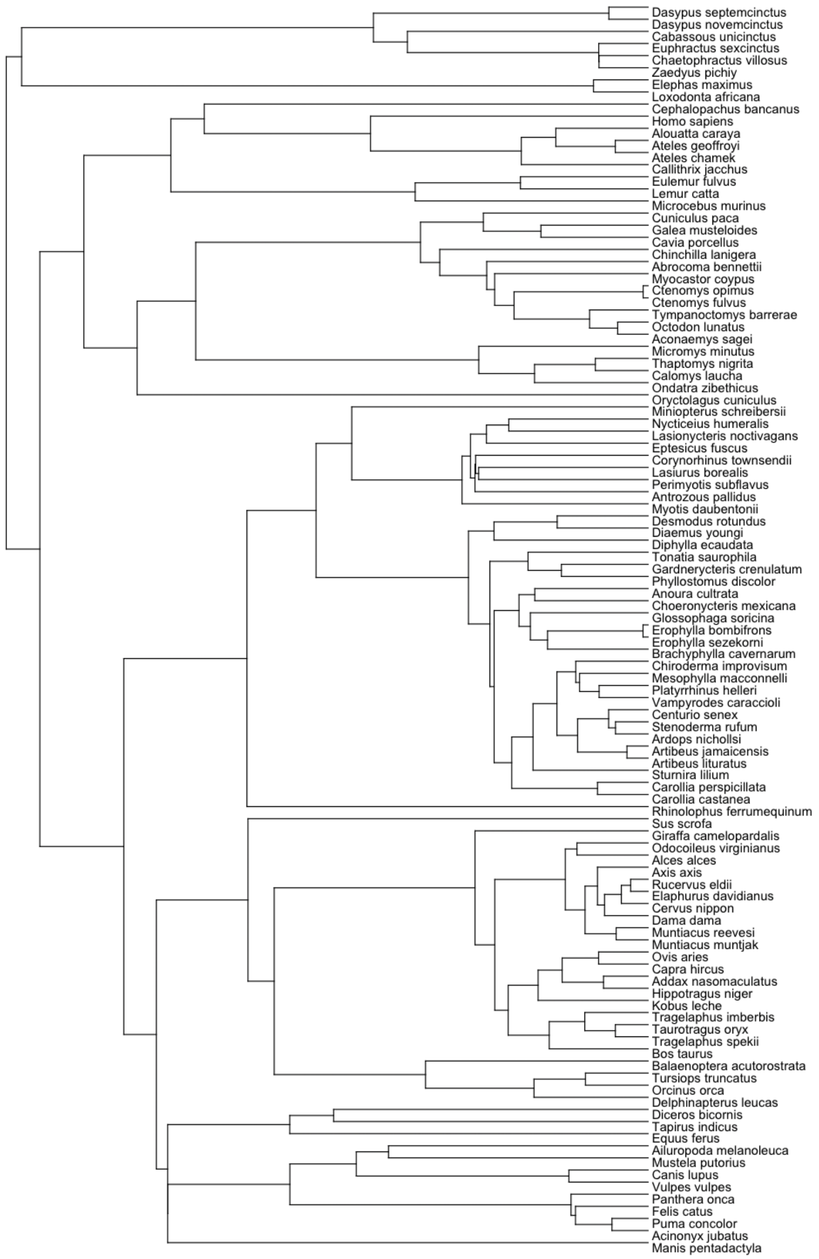

Fig. S2. Phylogenetic tree of sauropsid species in the database. Tip-to-root 279.8 MY. Generated using TimeTree v 5.0 (Kumar et al., 2022).

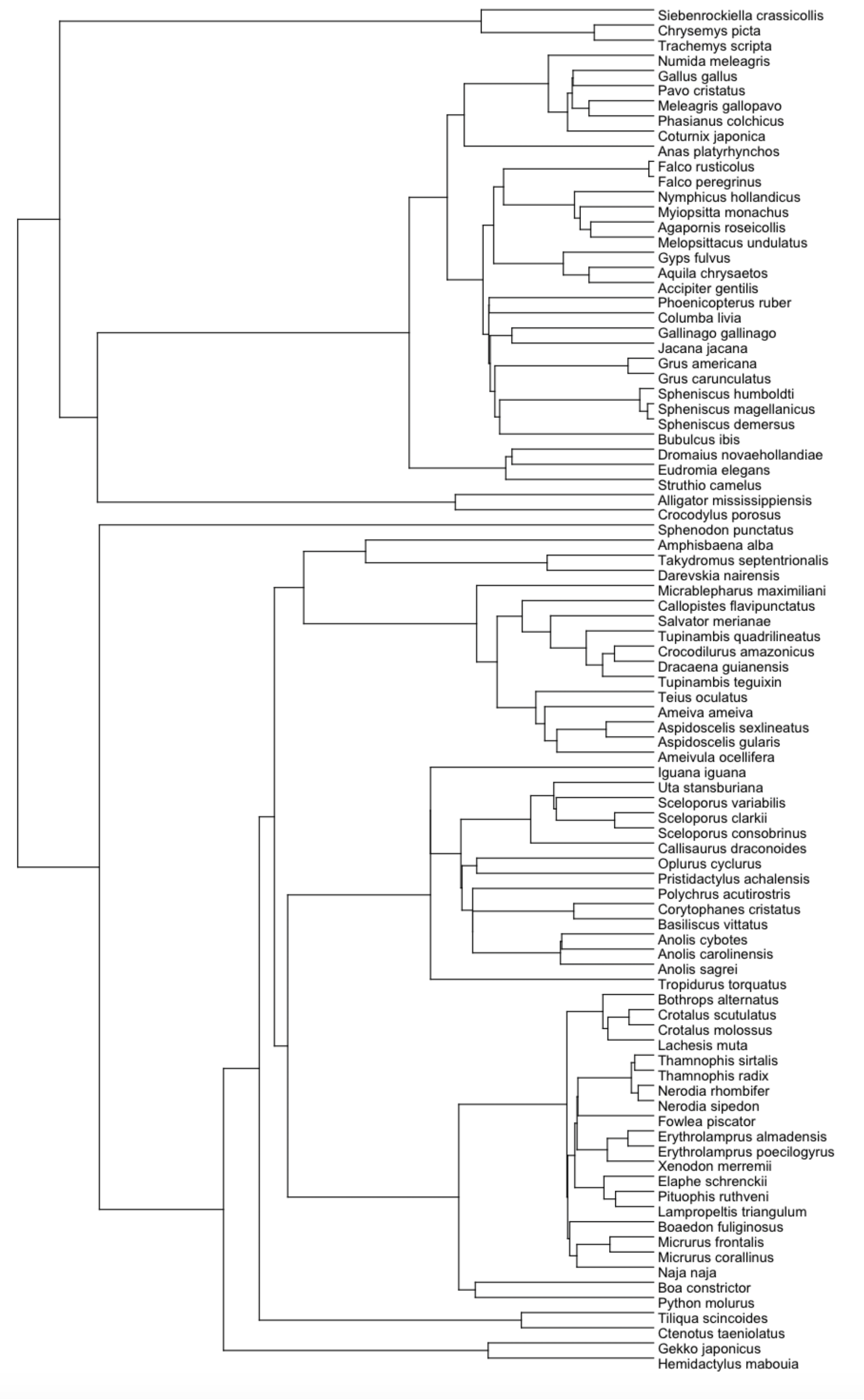

VII. Table S1. Database summary

	No. species	Head size	Dispersity K	Chromosome number 2n
Mammalia (spatulate)	103	Area 7.2 – 226 μm^2	1.7 – 16	6 – 102
Sauropsids (vermiform)	90	Length 6.3 – 59 µm	4.0 – 36	20 – 98
